# Patterns of change in the association between socioeconomic status and body mass index distribution in India, 1999–2021

**DOI:** 10.7189/jogh.14.04171

**Published:** 2024-10-11

**Authors:** Meekang Sung, Anoop Jain, Akhil Kumar, Rockli Kim, Bharati Kulkarni, S V Subramanian

**Affiliations:** 1 Department of Social and Behavioral Sciences, Harvard T. H. Chan School of Public Health, Boston, Massachusetts, USA; 2 Department of Environmental Health, Boston University School of Public Health, Boston, Massachusetts, USA; 3 Faculty of Arts and Sciences, University of Toronto, Toronto, Ontario, Canada; 4 Division of Health Policy and Management, College of Health Science, Korea University, Seoul, South Korea; 5 Interdisciplinary Program in Precision Public Health, Department of Public Health Sciences, Graduate School of Korea University, Seoul, South Korea; 6 Division of Reproductive & Child Health & Nutrition, Indian Council of Medical Research, V. Ramalingaswami Bhawan, Ansari Nagar, New Delhi, India; 7 Harvard Center for Population and Development Studies, 9 Bow Street Cambridge, Massachusetts, USA

## Abstract

**Background:**

Body mass index (BMI) is an important indicator of human health. However, trends in socioeconomic inequalities in BMI over time throughout India are understudied. Filling this gap will elucidate which socioeconomic groups are still at risk for adverse BMI values.

**Methods:**

This repeated cross-sectional study analysed four rounds of India’s National Family Health Surveys (1998–1999, 2005–2006, 2015–2016, and 2019–2021). The outcome was BMI categories, measured in kilogram per metres squared (kg/m^2^), defined as severely/moderately thin (<17.0 kg/m^2^), mildly thin (17.0–18.4 kg/m^2^), normal (18.5–24.9 kg/m^2^), overweight (25.0–29.9 kg/m^2^), and obese (≥30.0 kg/m^2^). We examined the prevalence, standardised absolute change, and odds ratios estimated by multivariable regression models by household wealth and levels of education, two important measures of socioeconomic status (SES).

**Results:**

The study population consisted of 1 244 149 women and 227 585 men. We found that those in the lowest SES categories were more likely to be severely/moderately thin or mildly thin. Conversely, those in the highest SES groups were more likely to be overweight or obese. The gradients were steepest for wealth, and this was substantiated by the results of regression models for every wave. There has been a decline in the difference in the prevalence of severely/moderately thin or mildly thin between SES groups when comparing the years 1999 and 2021.

**Conclusions:**

SES-based inequalities in BMI were smaller in 2021 compared to 1999. However, those in low SES groups were most likely to be severely/moderately thin or mildly thin while those in high SES groups were more likely to be overweight or obese. Future research should explore the pathways that link SES with BMI.

The double burden of obesity and undernutrition is becoming increasingly common in low- and middle-income countries [1]. In India, the share of people with a high body mass index (BMI) has steadily risen from 10% in 1999 to 23% in 2021 [2,3]. More than 135 million Indian adults were estimated to be obese in 2015 [4]. Factors associated with this increased prevalence of obesity include improved economic conditions, urbanisation, an increase in sedentary lifestyles, and dietary changes [5,6]. However, India also has the highest share of adults who are underweight [7]. This burden is particularly pronounced among women, with over 100 million adult women being underweight as of 2014, which accounted for 41.6% of the global underweight [7]. Poverty and consistent nutritional deficiencies are key determinants of low body weight in India [8].

A person’s BMI serves as an important indicator of health [9]. Individuals with a BMI over 25 kg/m^2^ are particularly susceptible to many non-communicable diseases such as hypertension, type 2 diabetes, and pulmonary illness [10,11], which, in turn, elevate mortality and disability rates [12] and impair overall quality of life [13]. Those with a BMI lower than 18.5 kg/m^2^ are at a greater risk for complications during pregnancy and poor birth outcomes [14], skeletal muscle wasting [15], a reduction in physical work capability, and consequently, a decrease in economic output [16].

Prior studies have shown that indicators of socioeconomic status (SES) are important predictors of BMI among Indian adults. There is an inverse relationship between SES and adult BMI in high-income countries, with low SES corresponding to higher BMI and vice versa [17]. In India, however, previous studies show that wealthier, more educated, higher caste, and urban-living adults are at a greater risk of being overweight or obese [18–22]. Those with higher SES are more likely to consume sugar-sweetened beverages and live sedentary lifestyles, both of which are factors associated with overweight and obesity [5,23–26]. In contrast, those in low SES categories are less likely to have diverse diets and high-quality proteins [26–28]. There is also growing recognition of obesity rising in low SES groups such as urban slum residents [29]. The full bibliography of the results of the systematic search on the topic is presented in Appendix S1 in the **Online Supplementary Document**.

What remains unknown, however, is how the double burden of malnutrition has changed over time by SES throughout India. Therefore, we examined trends in BMI by SES using data sets from four rounds (1999, 2006, 2016, 2021) of a nationally representative survey from India. The results from this study will provide key insights into understanding BMI distribution in relation to SES, thereby helping policymakers and programme implementers better understand how to best target interventions for more equitable nutrition outcomes throughout India.

## METHODS

### Data

The study used data from four waves of the National Family Health Survey (NFHS) in India [2,3,30,31]. The four surveys were conducted in 1998-99 (NFHS-2), 2005-06 (NFHS-3), 2015-16 (NFHS-4) and 2019-21 (NFHS-5). The most recent Census of India available at the time of the survey serves as the sample frame for all NFHS rounds (NFHS2: Census of India 1991, NFHS3: Census of India 2001, NFHS 4/NFHS 5: Census of India 2011). Each round of the NFHS uses a multistage stratified cluster sampling design (Appendix S2 in the **Online Supplementary Document**), which is described in full in the most recent NFHS report [3]. The NFHS surveys collect data through four different questionnaires: the biomarker questionnaire, the individual (women’s and men’s) questionnaire, and the household questionnaire. The women's questionnaire collected information from all usual resident women in the household who were aged 15–49 years old and consented to the interview. In contrast, the men's questionnaire was only administered to usual resident men aged 15–54 years old living in households selected for the state module, resulting in a difference in sample size between the two groups [3]. The biomarker questionnaire, which contains data for the height and weight for men and women, were administered to all women aged 15–49 years and men aged 15–54 years old who lived in households selected for the state module regardless of their usual resident status.

The responses from the biomarker questionnaire are included in the Household Member Recode data set and, for women interviewed, also in the Individual Recode data set. Information on a woman’s pregnancy status is recorded in the biomarker and women’s questionnaire whereas the date of the most recent childbirth is only captured in the women’s questionnaire. Education, age and residence data is included in both the individual and household questionnaires, whereas wealth data are only recorded in the household questionnaire. We utilised the Individual Recode data set rather than the Household Member Recode data set for socioeconomic variables, because individual responses are more reliable and ensure consistent data analysis. For men, BMI data from the Household Member Recode data set was merged with the Individual Recode data.

To ensure validity and manage the administration of training and fieldwork effectively, the survey was conducted in two phases. In each phase, fieldwork was organised in groups of five adjacent districts to facilitate close monitoring and supervision. This approach allowed for multiple levels of oversight to maintain the quality and accuracy of the fieldwork [3]. The measurements were done by trained health investigators.

### Study population

The study group comprised of adult women between the ages of 20 and 49 who were not pregnant at the time of the survey and had not given birth within the last two months, along with men aged 20 to 54 who lived in households selected for the state module. The minimum age of 20 was chosen because BMI is used to categorise weight status in adults starting at this age [32]. The upper limit of age was determined by the NFHS survey design.

Observations that were missing data on any anthropometric measurements of BMI (weight and height), had implausible BMI values (defined as BMI less than 12 or greater than 60), or were missing on any socioeconomic variables (household wealth and education) were excluded from our analysis. Since the NFHS-2 survey did not include BMI information of men, analysis for men was conducted for NFHS-3, NFHS-4, and NFHS-5 only. The final analytic sample was 71 356 women (1999), 90 33 women and 57 004 men (2006), 531 433 women and 90 227 men (2016), and 551 027 women and 80 354 men (2021) (**Table 1**).

**Table 1:** Study sample size selection from the four National Family Health Surveys (NFHS), 1999–2021

Survey round (year)	Sample size based on inclusion criteria (n)		Missing or implausible SES values (n)		Missing or implausible BMI values (n, %)		Final study sample size (n)	
	Women	Men	Women	Men	Women	Men	Women	Men
NFHS-2 (1998–99)	76 880	-*	33	-*	5491 (7.1)	-*	71 356	-*
NFHS-3 (2005–06)	94 575	61 291	0	25	4242 (4.5)	4262 (7.0)	90 333	57 004
NFHS-4 (2015–16)	540 840	93 040	0	0	9407 (1.7)	2813 (3.0)	531 433	90 227
NFHS-5 (2019–21)	569 203	85 182	0	0	18 176 (3.2)	4828 (5.7)	551 027	80 354
All waves†	1 281 498	239 513	33	25	37 316 (2.9)	11 903 (5.0)	1 244 149	227 585

BMI – body mass index, SES – socio-economic status

*Men data for NFHS-2 (1999) are indicated as ‘-’ (not applicable) since men samples were not included in the survey.

†The ‘overall’ or ‘pooled’ values.

### Outcome

Weight and height measurements for calculating BMI were taken using the Seca 874 digital scale and the Seca 213 stadiometer, respectively. The scale is a standard, self-calibrating device equipped with the latest technology to minimise instrument errors. Additionally, all equipment used in the survey was periodically standardised to ensure the accuracy and consistency of the measurements [33].

Based on the BMI cut-offs of WHO [32] and definitions of chronic energy deficiency of the International Dietary Energy Consultative Group [34], the BMI outcomes were divided into five categories: severely/moderately thin (<17.0 kg/m^2^), mildy thin (17.0–18.4 kg/m^2^), normal (18.5–24.9 kg/m^2^), overweight (25.0–29.9 kg/m^2^), and obese (≥30.0 kg/m^2^).

### Variables on socioeconomic status and demographics

We assessed changes in BMI outcomes over time by household wealth quintiles and educational attainment. We also assessed changes by age (categorised in five-year increments from 20 to 49 for women, and from 20 to 54 for men) and place of residence (rural/urban). The main findings focused on socioeconomic variables (wealth, education), while detailed analyses for age groups and place of residence are provided in the supplementary files.

The NFHS surveys record the number of years of education completed, corresponding to grade level and highest educational degree received. Based on this data, we created five education categories: a) no schooling (less than one year of schooling), b) 1st to 5th grade (one to five years of schooling, equivalent to finishing primary school), c) 6th to 8th grade (six to eight years of schooling, equivalent to finishing middle school), d) 9th to 12th grade (nine to twelve years of schooling, equivalent to finishing high school), and e) above 12th grade (more than 12 years, equivalent to undergraduate or graduate coursework). Hereafter education level, or educational attainment is referred to as the level of schooling.

The household wealth index is a composite measure of a household's overall living standard. In NFHS, it is calculated using data on household ownership of selected assets, such as televisions and bicycles, housing construction materials, and types of water access and sanitation facilities [35]. Detailed variables used to construct the household wealth index in India are available on the official Demographic Health Surveys (DHS) website [35]. Quintiles of household wealth index are constructed as lowest (bottom 20th percentile), low (20th to 40th percentile), middle (40th to 60th percentile), high (60th to 80th percentile), and highest (top 20th percentile).

For analysis done by place of residence, we used the definition of rural and urban defined by the India Office of the Registrar General and Census Commissioner, which is also officially used by NFHS. Urban areas included statutory towns and census towns. Statutory towns are places with substantive administration bodies (includes municipality, corporation, cantonment boards, and notified town area committee). Census towns are defined as having a minimum population of 5000, at least 75 percent of the male workforce engaged in non-agricultural jobs, and a population density of at least 400 per km^2^. All other areas not categorised as urban are considered as rural area [36].

### Analysis

We calculated the weighted prevalence and 95% confidence intervals for each BMI outcome in each period. We used the individual weights from the survey to account for the multi-stage stratified cluster sampling design. We also calculated the standardised absolute change (SAC) to quantify the extent of change in BMI outcomes across time periods. For example, SAC between 2016 and 2021 is computed as follows:


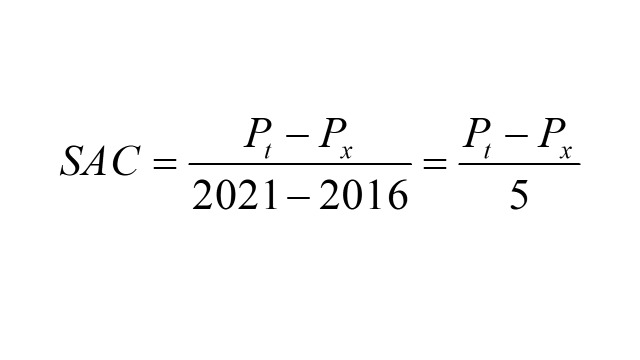
;

(*P_t_* = percentage at recent year, *P_x_* = percentage at a previous year in consideration)

A negative SAC value indicates a lowering of prevalence in BMI outcomes, whereas a positive SAC value indicates an increase of prevalence in BMI outcomes.

Finally, we estimated unadjusted, age-adjusted, and multivariable logistic regression to estimate the odds ratios and 95% confidence intervals to assess the independent associations between household wealth, education, and place of residence on each BMI outcome by survey year. The reference group in regression models were those in the lowest SES (lowest wealth quintile, no schooling), and rural for place of residence.

We used Stata 15.0 (2017, College Station, Texas, USA), Tableau (2016, Mountain View, California, USA), and Excel for the analysis and for constructing the visualisations.

## RESULTS

### Sample characteristics

Our study contained complete BMI and SES data from 1 244 149 non-pregnant women aged 20–49 from NFHS surveys in 1999, 2006, 2016, 2021 and 227 585 men aged 20–54 from NFHS surveys in 2006, 2016, 2021 (**Table 1**). The proportion of women with missing or implausible BMI data varied between 1.7% and 7.1%, while for men, it ranged from 3.0% to 7.0% (**Table 1**). Populations in the highest wealth quintiles, highest level of schooling, and rural residence had a bigger proportion of excluded or missing BMI values (Table S1 in the **Online Supplementary Document**).

The proportion of people with higher education increased between 1999 and 2021. For example, while in 1999, 49.6% of the women study population had no schooling, the proportion decreased substantially to 27.7% in 2021 (**Table 2**).

**Table 2:** Demographic and socioeconomic characteristics of the study population for men and women (1999–2021)

Variables	Women								Men					
	**1999**		**2006**		**2016**		**2021**		**2006**		**2016**		**2021**	
	**n**	**%**	**n**	**%**	**n**	**%**	**n**	**%**	**n**	**%**	**n**	**%**	**n**	**%**
Age, in years														
*20–24*	11 788	16.5	18 902	20.9	104 817	19.7	100 434	18.2	11 547	20.3	16 088	17.8	13 467	16.8
*25–29*	15 011	21.0	17 921	19.8	101 657	19.1	103 227	18.7	10 232	17.9	15 609	17.3	13 444	16.7
*30–34*	14 030	19.7	16 384	18.1	91 077	17.1	93 646	17.0	9040	15.9	14 210	15.7	12 447	15.5
*36–39*	12 518	17.5	15 233	16.9	87 515	16.5	93 553	17.0	8558	15.0	13 497	15.0	12 223	15.2
*40–44*	10 129	14.2	12 507	13.8	75 043	14.1	78 666	14.3	7274	12.8	11 612	12.9	10 280	12.8
*45–49*	7880	11.0	9386	10.4	71 324	13.4	81 501	14.8	6114	10.7	10 863	12.0	10 314	12.8
*50–54*									4239	7.4	8348	9.3	8179	10.2
Household wealth (quintile)														
*Lowest (bottom 20%)*	10 566	14.8	9940	11.0	97 291	18.3	109 910	20.0	5560	9.8	14 657	16.2	15 407	19.2
*Low*	11 186	15.7	12 539	13.9	110 180	20.7	119 828	21.8	7943	13.9	18 412	20.4	17 667	22.0
*Middle*	13 852	19.4	16 986	18.8	111 415	21.0	116 183	21.1	11 449	20.1	19 569	21.7	17 221	21.4
*High*	16 559	23.2	21 973	24.3	107 844	20.3	108 927	19.8	14 867	26.1	18 895	20.9	16 225	20.2
*Highest (top 20%)*	19 193	26.9	28 895	32.0	104 703	19.7	96 179	17.5	17 185	30.1	18 694	20.7	13 834	17.2
Levels of schooling														
*No schooling*	35 396	49.6	32 505	36.0	176 915	33.3	152 369	27.7	9345	16.4	13 982	15.5	11 163	13.9
*1st–5th grade*	11 950	16.7	13 094	14.5	72 771	13.7	72 176	13.1	9220	16.2	12 641	14.0	10 152	12.6
*6th–8th grade*	8744	12.3	12 733	14.1	79 918	15.0	85 018	15.4	9609	16.9	15 753	17.5	13 349	16.6
*9th–12th grade*	11 522	16.1	21 414	23.7	135 300	25.5	157 624	28.6	18 859	33.1	32 009	35.5	29 919	37.2
*>12th grade*	3744	5.2	10 587	11.7	66 529	12.5	83 840	15.2	9971	17.5	15 842	17.6	15 771	19.6
Place of residence														
*Rural*	47 992	67.3	49 004	54.2	372 697	70.1	412 314	74.8	28 401	49.8	61 811	68.5	59 713	74.3
*Urban*	23 364	32.7	41 329	45.8	158 736	29.9	138 713	25.2	28 603	50.2	28 416	31.5	20 641	25.7

### Changes in BMI outcomes over time

**Figure 1:**
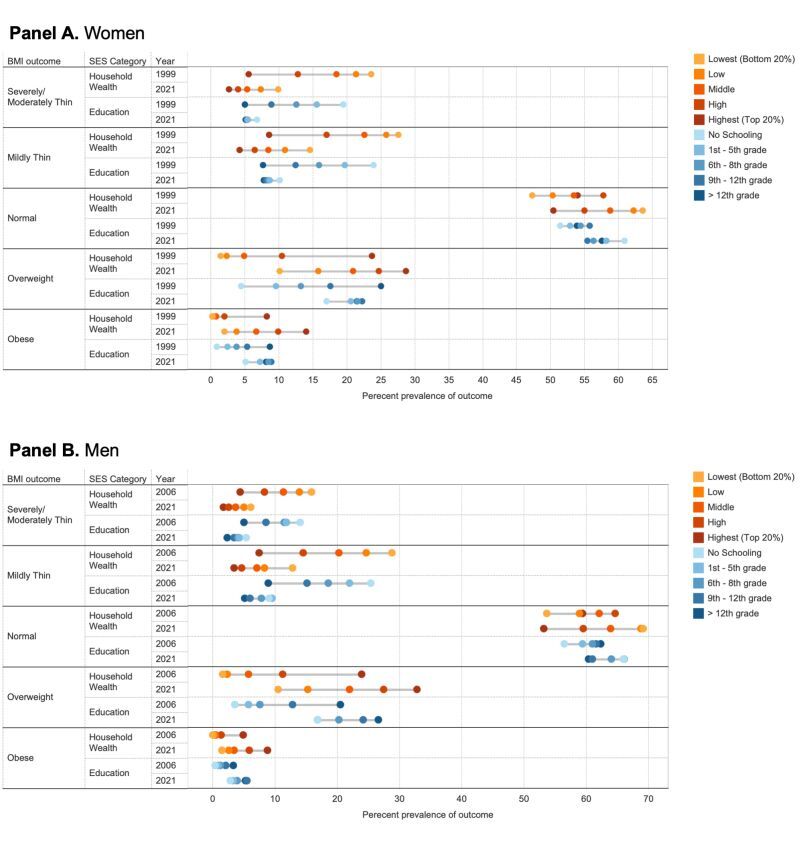
Trend of weighted prevalence of body mass index (BMI) categories by household wealth and levels of schooling. **Panel A.** Women. **Panel B.** Men. See Table S1 in the **Online Supplementary Document** for the corresponding table of percent prevalence of outcome.

The overall prevalence of severely/moderately thin and mildly thin decreased across all SES categories between 1999 to 2021 (**Figure 1**, Panels A**–**B, Figure S1 in the **Online Supplementary Document**). Severely/moderately thin and mildly thin were most common among younger adults, those from low-wealth quintile households, those with lower levels of education, and those who lived in rural areas.

Those in the lowest SES groups experienced the greatest reductions in the prevalence of severely/moderately thin and mildly thin between 1999 and 2021. The prevalence of severely/moderately thin in the lowest wealth quintile women decreased from 23.6% (95% CI = 22.9–24.3) in 1999 to 9.8% (95% CI = 9.7–10.0) in 2021. In contrast, prevalence decreased from 5.5% (95% CI = 5.1–5.9) to 2.6% (95% CI = 2.5–2.7) among highest wealth quintile women (Tables S2–3 in the **Online Supplementary Document**). This represents a relative reduction of 52% among the highest wealth quintile women, comparable to a 58% reduction among the lowest wealth quintile women.

The prevalence of overweight and obese increased in all SES categories from 1999 to 2021. For instance, the prevalence of obese in the no schooling group increased from 0.9% (95% CI = 0.8–1.0) in 1999 to 5.1% (95% CI = 5.0–5.2) in 2021. Older age adults, those in higher wealth quintile households, higher levels of schooling, and urban residence groups had a higher prevalence of overweight and obese in all time periods.

### Socioeconomic gaps in BMI outcomes

For all BMI outcomes, the wealth gradient was steeper than the education gradient. The prevalence gap of severely/moderately thin or mildly thin by wealth levels has decreased by around 10 percentage points during the earliest year (women = 1999, men = 2006) and 2021 (Figure S2 in the **Online Supplementary Document****)**. The change could be explained by the SAC over the years (**Table 2**). A bigger decline in severely/moderately thin and mildly thin percent prevalence occurred in the lowest quintile, with the biggest reduction in severely/moderately thin occurring between 2006 and 2016 (around −1 percentage points per year) (**Table 3**, Figure S3 in the **Online Supplementary Document**).

**Table 3:** Standardised absolute change of body mass index (BMI) categories by highest and lowest socioeconomic characteristics

BMI outcome	Severely/moderately thin			Mildly thin			Normal			Overweight			Obese		
**Year**	1999–2006	2006–2016	2016–2021	1999–2006	2006–2016	2016–2021	1999–2006	2006–2016	2016–2021	1999–2006	2006–2016	2016–2021	1999–2006	2006–2016	2016–2021
**Women**															
Household wealth (quintile)															
*Lowest (bottom 20%)*	0.03	−1.01	−0.78	0.05	−0.85	−0.97	−0.16	1.36	0.75	0.05	0.43	0.80	0.02	0.07	0.19
*Highest (top 20%)*	0.01	−0.26	−0.08	−0.04	−0.33	−0.14	−0.48	0.09	−0.24	0.25	0.26	0.12	0.25	0.23	0.34
Levels of schooling															
*No schooling*	−0.13	−0.89	−0.59	−0.21	−0.82	−0.80	−0.04	0.75	0.46	0.30	0.72	0.68	0.09	0.23	0.26
*>12th grade*	0.38	−0.21	−0.10	0.33	−0.15	−0.13	−0.02	0.46	−0.15	−0.37	−0.18	0.18	−0.32	0.08	0.19
Place of residence															
*Urban*	−0.16	−0.79	−0.52	−0.17	−0.80	−0.66	−0.03	0.70	0.13	0.29	0.68	0.72	0.08	0.21	0.34
*Rural*	−0.05	−0.47	−0.18	−0.13	−0.46	−0.27	−0.26	0.13	0.10	0.27	0.49	0.09	0.17	0.32	0.26
**Men**															
Household wealth (quintile)															
*Lowest (bottom 20%)*		−1.05	−1.10		1.36	0.38		0.38	1.01		0.05	0.19		−1.05	−1.10
*Highest (top 20%)*		−0.35	−0.11		−0.30	−0.64		0.66	0.46		0.22	0.36		−0.35	−0.11
Levels of schooling															
*No schooling*		−1.19	−0.89		0.95	−0.02		0.74	1.14		0.16	0.16		−1.19	−0.89
>12th grade		−0.29	−0.16		−0.17	−0.04		0.49	0.24		0.17	0.08		−0.29	−0.16
Place of residence															
*Urban*		−0.98	−0.75		0.57	−0.10		0.85	0.94		0.17	0.21		−0.98	−0.75
*Rural*		−0.56	−0.27		−0.18	−0.14		0.91	0.35		0.25	0.23		−0.56	−0.27

The education gradient for overweight and obese decreased by approximately 10 percentage points during the earliest year and 2021 for both genders. A slight increase of severely/moderately thin (+0.38 percentage points) and mildly thin (+0.33 percentage points) in the group with the highest level of schooling in 1999–2006 is also notable (**Table 3**). The difference also decreased in overweight and obese **(**Figure S2 in the **Online Supplementary Document****)**. The large increase in the percent prevalence of overweight in the population with no schooling explains the change. The group with the highest education had a notable decrease in obese prevalence in women from 1999–2006 (−0.32 percentage points) for women and 2006–2016 (−0.29 percentage points) for men (**Table 3**).

### Regression based inferences

**Figure 2:**
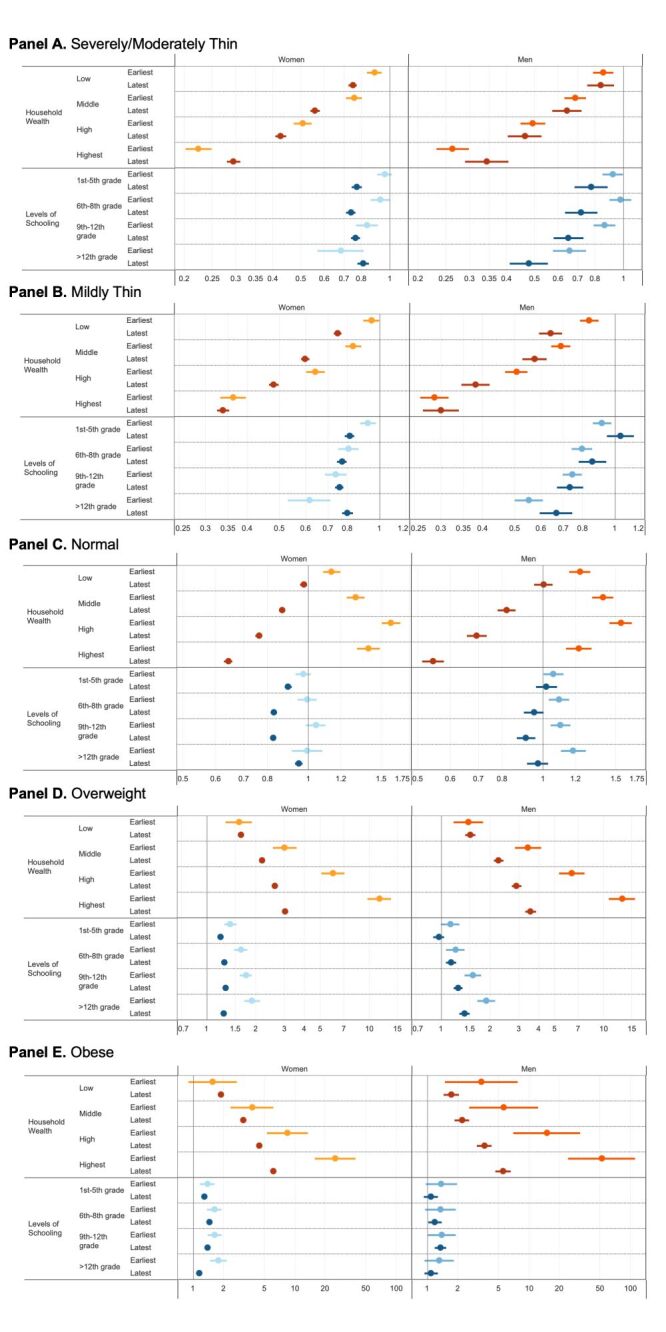
Adjusted odds ratio and 95% confidence interval for body mass index outcome by year. **Panel A.** Severely/moderately thin. **Panel B.** Mildly thin. **Panel C.** Normal **Panel D.** Overweight. **Panel E.** Obese. The reference group for household wealth (‘wealth’ in legend) is ‘lowest (bottom 20%)’. The reference group for levels of schooling is ‘no schooling’. The reference group for residence is ‘rural’. The axis is logarithmically scaled. The earliest year for women is 1999 (wealth – light orange, wealth – light blue) and 2006 (wealth – dark orange, levels of schooling – medium blue) for men. The latest year is 2021 (wealth – dark red, levels of schooling – dark blue) for both women and men. The darker colour of each socioeconomic outcome indicates more recent years; for example, the lightest green for wealth is 1999 and the darkest green for wealth is 2021.

Adults in the lowest SES categories for wealth, education, and residence had higher odds of severely/moderately thin and mildly thin, when compared to adults in the highest SES categories, in each of the four time periods. The association between SES and BMI outcomes was stronger for household wealth than schooling or residence. For example, the OR for severely/moderately thin was smallest in the highest group (reference: lowest group) in 2006 for women, 0.21 (95% CI = 0.19–0.23) (Table S4 in the **Online Supplementary Document****)**. The OR slightly increased towards 1 (null) for severely/moderately thin in the ‘high’ and ‘highest’ (top 20%) group during 1999–2021 (**Figure 2**, Panel A).

The pattern of adults with higher levels of schooling being less likely to experience severely/moderately thin and mildly thin was most clearly demonstrated in the most educated group (above 12th grade) in men for severely/moderately thin in 2021 (**Figure 2**, Panels A–B), where OR = 0.48 (95% CI = 0.41–0.55) (Table S4 in the **Online Supplementary Document**).

In contrast, people in the highest SES categories for wealth, education, and residence had higher odds of being overweight and obese, when compared to adults in the lowest SES categories. Similarly, OR contrast was most prominent wealth categories, indicating a higher likelihood of high SES individuals being overweight or obese. The high OR has reduced in the recent NFHS surveys. For instance, the OR of obese in the highest wealth quntile (top 20%) women population has fallen from 24.76 (95% CI = 15.62–39.26) in 1999 to 6.10 (95% CI = 5.78–6.44) in 2021 (Table S4 in the **Online Supplementary Document**).

## DISCUSSION

This study had four salient findings. First, by 2021, the prevalence of severely/moderately thin and mildly thin was higher than in preceding years. The prevalence of overweight and obesity was lower in 2021 compared to preceding years. Second, being severely/moderately thin or mildly thin was more likely among individuals in the lowest SES categories, while being overweight or obese was more likely among those in the highest SES categories. Third, SES-based inequalities of severely/moderately thin and mildly thin were smaller in 2021 than in the previous NFHS survey years. Those in the lowest SES groups experienced the largest absolute decreases in the prevalence of severely/moderately thin and mildly thin. Fourth, the SES gradients were steepest for wealth, and this was substantiated by the results of regression models.

The following data-related issues should be considered while interpreting the findings. First, NFHS-2 (1998–99) did not collect any data from men, making the comparison between men and women over all time periods difficult. Second, NFHS-5 was initiated in 2019 and was disrupted due to COVID-19 pandemic. Hence its completion was delayed until 2021 [3]. More research is required to assess any systematic influences of the pandemic on our outcomes of interest. Third, our outcome measures derived solely from BMI have limitations in understanding body composition or health risks. Ideally, waist circumference and body fat percentage should also be considered [37]. Restricting study outcome to BMI was due to the availability, comparability, and validity of data across NFHS surveys. Fourth, the excluded study populations due to missing or implausible BMI values had a higher proportion of individuals from the highest wealth or most educated groups, potentially introducing estimation errors to our findings. However, since the proportion of missing data are small (<5%), it is unlikely to have significantly altered the results. Fifth, variables on the level of schooling and age were based on self-reports of individuals, which may have measurement errors differential by gender or other confounders. However, given the large sample size, the descriptive nature of the study, and the various steps taken by the NFHS for data quality management, the errors are unlikely to significantly alter study findings. Sixth, our paper did not analyse the heterogeneity of the association within states. Further research on the subnational variation is needed.

The results of this study are consistent with previous studies that show that being underweight is more common among those in the lowest SES categories [38] and that being overweight is more prevalent among more educated and wealthier Indian adults [39-41]. This study shows that this pattern has not changed over the four rounds of cross-sectional surveys (1999, 2006, 2016, 2021). Our findings also show decreasing inequalities in BMI outcomes throughout India. Trends of decreasing overweight gaps between SES groups have been observed globally, driven by increasing prevalence in lower SES groups [42-45].

India's unique pattern of BMI and SES can be interpreted through Omran’s epidemiologic transition theory, which posits a long-term shift in mortality and disease patterns where pandemics of infectious diseases are gradually replaced by ‘degenerative and man-made diseases’ [46]. Although Omran did not explicitly define ‘degenerative and man-made diseases’, he later cited cardiovascular disease, cancer, stroke, diabetes, and metabolic disorders as examples. The rise in overweight and obesity across all SES, particularly among low SES populations, exemplifies the increasing burden of non-communicable diseases in India [47].

However, applying the epidemiologic transition theory should be approached cautiously, as Omran did not focus directly on the rise of chronic diseases; his theory was closely tied to efforts to accelerate fertility decline through health-oriented population control programmes [48]. Additionally, the theory's assumption of population homogeneity does not reflect the reality of diverse populations, where infectious diseases remain prevalent among the poor while non-communicable diseases are common among the rich [49], leading to a ‘double burden’ of disease in many low- and middle- income countries including India.

Empirical evidence suggests that some unconsidered aspects of social determinants contributed to deviations from classic epidemiologic transition [50]. Studies conducted in India have identified higher SES as an important factor involved in access to high-calorie food and physical inactivity, factors associated with a higher body weight [39]. Changes in the socioeconomic environment for low SES individuals may contribute to deviation from the existing pattern to the rapid rise in overweight/obesity and the decline in underweight among low SES groups. The decreasing cost of high-calorie, less nutritious, and highly processed foods and beverages may influence eating habits [22,51]. Some researchers suggest that exposure to high-calorie food have a stronger effect in low-income or rural settings due to the more favourable perception of larger body sizes [52,53]. Additionally, lower SES individuals in urban areas may be less likely to live in walkable communities with green spaces, contributing to declining rates of physical activity [24,54]. Conversely, increased health knowledge and the financial ability to afford low-calorie diets may explain the smaller change in overweight/obesity prevalence among higher SES populations [22,55,56].

Robust policies in India on food deprivation and insecurity, such as the Targeted Public Distribution System [57] of 1997 and the National Food Security Act on legal subsidies on food [58], may have contributed to a decline in underweight prevalence, especially in low SES groups. Despite these efforts, those in the lowest SES strata continued to be most likely to have the highest prevalence of underweight. This warrants that marginalised populations continue to be prioritised by efforts aimed at lowering the burden of underweight. Also, the mechanisms of the distribution change of BMI outcomes need to be further clarified to determine effective and precise policy interventions in the social environment on undernutrition and obesity.

## CONCLUSIONS

Our results show that lower SES individuals are consistently more likely to experience low BMI while higher SES individuals have increased odds of high BMI. Inequalities in BMI distribution across SES in BMI have decreased in 2021 compared to earlier years indicating improved nutrition security for the low SES population groups. However, the convergence could be due to different mechanisms by high and low-SES individuals and their associated social environment.

## Additional material


Online Supplementary Document
