# Supplementary material for: Patterns of change in the association between socioeconomic status and body mass index distribution in India, 1999–2021
Source: J Glob Health. 2024 Oct 11;14:04171. doi: 10.7189/jogh.14.04171 (PMC11467771; doi:10.7189/jogh.14.04171)
Supplement: Online Supplementary Document [file jogh-14-04171-s001.pdf]

Table S1: Distribution Comparison of Sociodemographic Characteristics of Study Population versus Sample Population Missing Plausible BMI Values

	Women		Men	
**Population**	Study (n)	Missing BMI (n, %*)	Study (n)	Missing BMI (n, %)
**Age (in years)**				
20-24	235,941	7,971 (3.3)	41,102	2,397 (5.5)
25-29	237,816	7,216 (2.9)	39,285	2,277 (5.5)
30-34	215,137	6,185 (2.8)	35,697	1,994 (5.3)
36-39	208,819	6,038 (2.8)	34,278	1,631 (4.5)
40-44	176,345	5,082 (2.8)	29,166	1,417 (4.6)
45-49	170,091	4,824 (2.8)	27,291	1,252 (4.4)
50-54			20,766	935 (4.3)
**Household Wealth (Quintile)**				
Lowest (Bottom 20%)	227,707	5,352 (2.3)	35,624	1,401 (3.8)
Low	253,733	5,690 (2.2)	44,022	1,792 (3.9)
Middle	258,436	6,202 (2.3)	48,239	2,143 (4.3)
High	255,303	7,633 (2.9)	49,987	2,790 (5.3)
Highest (Top 20%)	248,970	12,439 (4.8)	49,713	3,777 (7.1)
**Levels of Schooling**				
No Schooling	397,185	12,017 (2.9)	34,490	1,734 (4.8)
1st-5th grade	169,991	3,940 (2.3)	32,013	1,430 (4.3)
6th-8th grade	186,413	4,406 (2.3)	38,711	1,819 (4.5)
9th-12th grade	325,860	8,997 (2.7)	80,787	4,181 (4.9)
>12th grade	164,700	7,956 (4.6)	41,584	2,739 (6.2)
**Place of Residence**				
Rural	362,142	16,518 (4.4)	77,660	5,950 (7.1)
Urban	882,007	20,798 (2.3)	149,925	5,953 (3.8)

*Percentages were calculated as missing/(missing + included BMI) for each socioeconomic and demographic group.

Table S2: Weighted Prevalence of Body Mass Index (BMI) categories by SES Characteristics, Women (A) and Men (B)

A. Women, 1999-2021

	Severely/Moderately Thin				Mildly Thin				Normal				Overweight				Obese			
	1999	2006	2016	2021	1999	2006	2016	2021	1999	2006	2016	2021	1999	2006	2016	2021	1999	2006	2016	2021
**India**	15.9 (15.7-16.2)	14.6 (14.3-14.8)	7.6 (7.5-7.7)	5.7 (5.6-5.7)	19.9 (19.6-20.2)	18.2 (18.0-18.5)	11.2 (11.1-11.3)	8.7 (8.6-8.8)	52.7 (52.4-53.1)	52.0 (51.7-52.4)	57.0 (56.9-57.1)	57.8 (57.6-57.9)	9.0 (8.8-9.2)	11.7 (11.5-11.9)	18.1 (18.0-18.2)	20.4 (20.3-20.5)	2.4 (2.3-2.6)	3.5 (3.4-3.6)	6.1 (6.0-6.1)	7.5 (7.4-7.6)
**Age (in years)**																				
20-24	17.9 (17.2-18.6)	17.0 (16.4-17.5)	11.8 (11.6-12.0)	10.4 (10.2-10.6)	25.6 (24.8-26.3)	23.8 (23.2-24.4)	17.4 (17.2-17.7)	15.0 (14.8-15.2)	53.3 (52.4-54.1)	53.4 (52.7-54.1)	60.7 (60.4-61.0)	62.4 (62.1-62.7)	2.9 (2.6-3.2)	5.0 (4.7-5.3)	8.1 (8.0-8.3)	9.5 (9.3-9.7)	0.4 (0.3-0.5)	0.9 (0.7-1.0)	2.0 (1.9-2.1)	2.7 (2.6-2.8)
25-29	16.9 (16.3-17.5)	15.8 (15.3-16.3)	8.4 (8.3-8.6)	6.5 (6.4-6.7)	22.8 (22.1-23.4)	19.5 (19.0-20.1)	12.8 (12.6-13.0)	10.5 (10.3-10.6)	53.1 (52.3-53.9)	54.0 (53.3-54.7)	60.3 (60.0-60.6)	61.5 (61.2-61.8)	6.1 (5.7-6.4)	8.7 (8.3-9.1)	14.6 (14.4-14.8)	16.8 (16.5-17.0)	1.2 (1.0-1.4)	2.0 (1.8-2.2)	3.9 (3.7-4.0)	4.8 (4.6-4.9)
30-34	15.2 (14.6-15.8)	14.7 (14.2-15.3)	6.7 (6.5-6.8)	4.7 (4.5-4.8)	19.7 (19.1-20.4)	18.2 (17.6-18.8)	10.2 (10.0-10.4)	7.6 (7.5-7.8)	53.2 (52.4-54.1)	51.8 (51.1-52.6)	57.3 (57.0-57.6)	57.8 (57.5-58.1)	9.4 (8.9-9.9)	12.1 (11.6-12.7)	19.6 (19.4-19.9)	22.2 (21.9-22.5)	2.4 (2.2-2.7)	3.1 (2.9-3.4)	6.2 (6.0-6.3)	7.7 (7.5-7.8)
36-39	14.4 (13.8-15.1)	13.2 (12.7-13.8)	5.8 (5.7-6.0)	4.0 (3.8-4.1)	17.0 (16.3-17.7)	15.7 (15.2-16.3)	8.6 (8.4-8.8)	6.5 (6.3-6.6)	53.6 (52.7-54.5)	51.4 (50.6-52.2)	56.0 (55.7-56.3)	55.6 (55.2-55.9)	11.5 (11.0-12.1)	15.0 (14.4-15.6)	21.7 (21.5-22.0)	24.5 (24.3-24.8)	3.4 (3.1-3.8)	4.6 (4.3-5.0)	7.8 (7.6-8.0)	9.5 (9.3-9.7)
40-44	15.0 (14.3-15.7)	12.6 (12.1-13.2)	5.9 (5.7-6.0)	3.6 (3.5-3.7)	16.5 (15.7-17.2)	14.4 (13.8-15.1)	8.1 (8.0-8.3)	5.7 (5.5-5.9)	51.2 (50.2-52.1)	49.9 (49.1-50.8)	52.9 (52.5-53.2)	53.9 (53.5-54.2)	13.5 (12.9-14.2)	16.7 (16.1-17.4)	24.0 (23.7-24.3)	26.0 (25.7-26.3)	3.9 (3.5-4.3)	6.3 (5.9-6.7)	9.2 (8.9-9.4)	10.8 (10.6-11.1)
45-49	15.7 (14.9-16.6)	12.0 (11.3-12.6)	5.6 (5.5-5.8)	3.9 (3.8-4.1)	14.1 (13.4-14.9)	13.7 (13.0-14.4)	7.7 (7.5-7.9)	5.5 (5.3-5.6)	50.8 (49.6-51.9)	49.7 (48.7-50.7)	52.3 (51.9-52.6)	53.6 (53.3-53.9)	14.5 (13.7-15.3)	18.2 (17.5-19.0)	24.9 (24.6-25.2)	26.1 (25.8-26.4)	4.9 (4.4-5.4)	6.5 (6.0-7.0)	9.5 (9.3-9.8)	10.9 (10.7-11.1)
**Household Wealth (Quintile)**																				
Lowest (Bottom 20%)	23.6 (22.9-24.3)	23.8 (23.2-24.5)	13.7 (13.5-13.9)	9.8 (9.7-10.0)	27.6 (26.8-28.3)	27.9 (27.2-28.6)	19.4 (19.1-19.7)	14.6 (14.3-14.8)	47.3 (46.5-48.2)	46.2 (45.4-47.0)	59.8 (59.5-60.1)	63.6 (63.3-63.9)	1.4 (1.2-1.6)	1.7 (1.5-2.0)	6.1 (5.9-6.2)	10.1 (9.9-10.3)	0.2 (0.1-0.2)	0.3 (0.2-0.4)	1.0 (0.9-1.0)	1.9 (1.9-2.0)
Low	21.3 (20.6-22.0)	20.8 (20.1-21.4)	10.6 (10.4-10.7)	7.3 (7.2-7.5)	25.8 (25.0-26.5)	24.3 (23.7-25.0)	15.2 (14.9-15.4)	10.9 (10.7-11.1)	50.3 (49.5-51.2)	50.3 (49.5-51.0)	60.6 (60.3-60.9)	62.2 (61.9-62.5)	2.3 (2.1-2.6)	4.1 (3.8-4.4)	11.4 (11.2-11.6)	15.8 (15.5-16.0)	0.3 (0.2-0.4)	0.6 (0.5-0.7)	2.2 (2.1-2.3)	3.8 (3.7-3.9)
Middle	18.5 (17.8-19.1)	15.7 (15.2-16.3)	7.4 (7.2-7.6)	5.3 (5.2-5.5)	22.6 (21.9-23.3)	20.0 (19.4-20.6)	11.2 (11.0-11.4)	8.4 (8.2-8.6)	53.5 (52.6-54.3)	55.3 (54.6-56.0)	59.3 (59.0-59.6)	58.7 (58.4-59.0)	4.8 (4.5-5.2)	7.8 (7.5-8.3)	17.6 (17.3-17.8)	20.9 (20.7-21.2)	0.7 (0.6-0.9)	1.2 (1.0-1.3)	4.5 (4.4-4.7)	6.6 (6.5-6.8)
High	12.8 (12.2-13.3)	10.4 (10.0-10.9)	5.1 (5.0-5.2)	4.0 (3.9-4.1)	17.0 (16.4-17.7)	14.3 (13.8-14.8)	7.7 (7.5-7.8)	6.5 (6.3-6.6)	57.8 (57.0-58.6)	56.7 (56.0-57.4)	54.9 (54.6-55.2)	55.0 (54.7-55.2)	10.4 (9.9-10.9)	15.1 (14.6-15.6)	23.8 (23.5-24.0)	24.7 (24.5-25.0)	2.0 (1.8-2.2)	3.6 (3.3-3.8)	8.6 (8.4-8.7)	9.9 (9.7-10.1)
Highest (Top 20%)	5.5 (5.1-5.9)	5.6 (5.3-5.9)	3.0 (2.9-3.1)	2.6 (2.5-2.7)	8.6 (8.1-9.0)	8.3 (7.9-8.7)	4.9 (4.8-5.1)	4.2 (4.1-4.4)	54.0 (53.2-54.8)	50.6 (50.0-51.3)	51.6 (51.3-51.9)	50.4 (50.1-50.7)	23.7 (23.1-24.4)	25.5 (24.9-26.1)	28.1 (27.8-28.4)	28.7 (28.4-29.0)	8.2 (7.8-8.7)	10.0 (9.6-10.4)	12.3 (12.2-12.5)	14.0 (13.8-14.3)
**Levels of Schooling**																				
No Schooling	19.5 (19.1-19.9)	18.6 (18.2-19.0)	9.7 (9.6-9.9)	6.8 (6.6-6.9)	23.8 (23.4-24.3)	22.3 (21.9-22.7)	14.1 (14.0-14.3)	10.1 (10.0-10.3)	51.4 (50.9-51.9)	51.1 (50.6-51.6)	58.6 (58.4-58.9)	60.9 (60.7-61.2)	4.4 (4.2-4.6)	6.5 (6.2-6.7)	13.6 (13.5-13.8)	17.0 (16.9-17.2)	0.9 (0.8-1.0)	1.5 (1.4-1.6)	3.8 (3.7-3.9)	5.1 (5.0-5.2)
1st-5th grade	15.6 (14.9-16.2)	13.7 (13.1-14.3)	8.1 (7.9-8.3)	5.4 (5.2-5.5)	19.6 (18.9-20.4)	17.8 (17.2-18.5)	11.7 (11.4-11.9)	8.6 (8.4-8.8)	52.9 (52.0-53.8)	53.5 (52.7-54.4)	56.5 (56.1-56.8)	58.2 (57.9-58.6)	9.5 (9.0-10.1)	11.7 (11.1-12.2)	18.0 (17.7-18.3)	20.6 (20.3-20.9)	2.4 (2.2-2.7)	3.3 (3.0-3.6)	5.8 (5.6-6.0)	7.2 (7.0-7.4)
6th-8th grade	12.6 (11.9-13.4)	12.7 (12.2-13.4)	7.1 (7.0-7.3)	5.3 (5.1-5.4)	15.9 (15.1-16.7)	16.3 (15.6-17.0)	10.4 (10.2-10.6)	8.4 (8.2-8.6)	54.4 (53.3-55.5)	51.6 (50.7-52.5)	55.7 (55.3-56.0)	56.3 (56.0-56.7)	13.2 (12.5-14.0)	14.4 (13.7-15.0)	19.8 (19.5-20.1)	21.6 (21.3-21.9)	3.8 (3.4-4.3)	4.9 (4.6-5.4)	7.0 (6.8-7.2)	8.4 (8.2-8.6)
9th-12th grade	8.9 (8.4-9.5)	9.8 (9.4-10.3)	6.0 (5.9-6.2)	5.3 (5.2-5.4)	12.4 (11.7-13.0)	13.4 (12.9-14.0)	9.2 (9.0-9.3)	8.1 (8.0-8.3)	55.8 (54.8-56.7)	52.7 (51.9-53.4)	55.3 (55.0-55.5)	55.4 (55.1-55.6)	17.6 (16.8-18.3)	18.0 (17.4-18.6)	21.6 (21.3-21.8)	22.2 (22.0-22.5)	5.3 (4.9-5.8)	6.1 (5.8-6.5)	7.9 (7.8-8.1)	8.9 (8.8-9.1)
>12th grade	5.0 (4.3-5.8)	7.7 (7.1-8.3)	5.5 (5.4-5.7)	5.1 (4.9-5.2)	7.6 (6.7-8.6)	10.0 (9.3-10.7)	8.4 (8.2-8.6)	7.8 (7.6-8.0)	53.8 (52.0-55.6)	53.6 (52.5-54.8)	58.2 (57.9-58.6)	57.5 (57.2-57.8)	25.0 (23.5-26.6)	22.4 (21.5-23.4)	20.6 (20.3-20.9)	21.5 (21.2-21.8)	8.6 (7.6-9.6)	6.3 (5.8-6.9)	7.2 (7.0-7.4)	8.1 (8.0-8.3)
**Place of Residence**																				
Rural	18.3 (18.0-18.7)	17.2 (16.9-17.5)	9.3 (9.2-9.4)	6.7 (6.6-6.7)	22.8 (22.4-23.2)	21.6 (21.3-21.9)	13.6 (13.5-13.7)	10.3 (10.2-10.4)	52.4 (52.0-52.9)	52.2 (51.8-52.6)	59.3 (59.1-59.4)	59.9 (59.7-60.1)	5.4 (5.2-5.6)	7.4 (7.2-7.7)	14.2 (14.1-14.3)	17.8 (17.7-17.9)	1.0 (0.9-1.1)	1.6 (1.5-1.7)	3.6 (3.6-3.7)	5.3 (5.3-5.4)
Urban	9.7 (9.3-10.1)	9.3 (9.0-9.6)	4.6 (4.5-4.7)	3.7 (3.6-3.7)	12.3 (11.8-12.7)	11.4 (11.0-11.7)	6.8 (6.7-6.9)	5.5 (5.4-5.6)	53.4 (52.7-54.1)	51.6 (51.1-52.2)	52.9 (52.6-53.1)	53.4 (53.1-53.6)	18.5 (17.9-19.0)	20.3 (19.9-20.8)	25.2 (25.0-25.4)	25.7 (25.5-25.9)	6.2 (5.8-6.5)	7.3 (7.1-7.7)	10.5 (10.4-10.7)	11.8 (11.7-12.0)

B. Men, 2006-2021

	Severely/Moderately Thin			Mildly Thin			Normal			Overweight			Obese		
	**2006**	**2016**	**2021**	**2006**	**2016**	**2021**	**2006**	**2016**	**2021**	**2006**	**2016**	**2021**	**2006**	**2016**	**2021**
**India**	10.2 (10.0-10.5)	4.9 (4.8-5.1)	3.7 (3.6-3.8)	18.2 (17.9-18.5)	9.8 (9.6-10.0)	7.0 (6.8-7.2)	60.2 (59.8-60.6)	63.1 (62.8-63.5)	62.7 (62.3-63.0)	9.8 (9.6-10.1)	18.6 (18.4-18.9)	22.1 (21.8-22.4)	1.6 (1.5-1.7)	3.5 (3.4-3.6)	4.5 (4.4-4.7)
**Age (in years)**															
20-24	12.2 (11.7-12.8)	7.4 (7.0-7.8)	6.9 (6.5-7.4)	23.2 (22.4-24.0)	15.1 (14.5-15.6)	11.7 (11.2-12.3)	59.8 (58.9-60.7)	66.4 (65.7-67.2)	68.0 (67.2-68.8)	4.1 (3.8-4.5)	9.6 (9.1-10.1)	11.0 (10.5-11.6)	0.6 (0.4-0.7)	1.5 (1.3-1.7)	2.3 (2.1-2.6)
25-29	10.0 (9.5-10.6)	4.7 (4.3-5.0)	4.0 (3.6-4.3)	20.3 (19.5-21.1)	10.2 (9.7-10.7)	7.9 (7.5-8.4)	61.0 (60.1-62.0)	66.8 (66.0-67.5)	65.8 (65.0-66.7)	7.7 (7.2-8.2)	15.7 (15.1-16.3)	18.6 (17.9-19.2)	0.9 (0.8-1.1)	2.7 (2.5-3.0)	3.7 (3.4-4.1)
30-34	8.7 (8.1-9.2)	4.1 (3.8-4.5)	2.8 (2.5-3.1)	17.2 (16.5-18.0)	8.2 (7.7-8.7)	6.1 (5.6-6.5)	62.1 (61.1-63.1)	64.6 (63.8-65.4)	61.9 (61.1-62.8)	10.3 (9.7-11.0)	19.5 (18.8-20.2)	24.4 (23.6-25.2)	1.6 (1.3-1.8)	3.6 (3.3-3.9)	4.8 (4.4-5.2)
36-39	8.7 (8.1-9.3)	4.0 (3.7-4.4)	2.4 (2.2-2.7)	16.1 (15.3-16.8)	7.9 (7.5-8.4)	6.3 (5.9-6.7)	60.8 (59.8-61.8)	61.7 (60.8-62.5)	60.5 (59.6-61.4)	12.2 (11.5-12.9)	22.2 (21.5-22.9)	25.4 (24.6-26.2)	2.0 (1.8-2.3)	4.2 (3.9-4.6)	5.4 (5.0-5.8)
40-44	9.9 (9.3-10.6)	4.3 (4.0-4.7)	3.0 (2.7-3.4)	15.5 (14.7-16.3)	7.9 (7.4-8.4)	5.3 (4.9-5.7)	59.8 (58.7-60.9)	60.0 (59.1-60.9)	60.1 (59.2-61.1)	12.6 (11.8-13.3)	23.1 (22.4-23.9)	26.0 (25.1-26.8)	2.1 (1.8-2.5)	4.6 (4.2-5.0)	5.6 (5.1-6.0)
45-49	11.1 (10.3-11.9)	4.5 (4.1-4.9)	3.1 (2.8-3.5)	16.1 (15.2-17.0)	8.4 (7.9-9.0)	4.8 (4.4-5.2)	57.2 (56.0-58.4)	59.4 (58.4-60.3)	60.0 (59.0-60.9)	13.3 (12.5-14.1)	23.3 (22.5-24.1)	26.6 (25.8-27.5)	2.5 (2.1-2.9)	4.4 (4.1-4.8)	5.6 (5.1-6.0)
50-54	11.0 (10.1-12.0)	4.9 (4.5-5.4)	3.0 (2.7-3.4)	15.0 (14.0-16.1)	9.4 (8.8-10.1)	5.7 (5.2-6.2)	57.5 (56.0-58.9)	58.5 (57.4-59.5)	60.2 (59.2-61.3)	14.0 (13.0-15.1)	22.2 (21.3-23.1)	26.0 (25.1-27.0)	2.4 (2.0-2.9)	5.0 (4.5-5.5)	5.1 (4.6-5.6)
**Household Wealth (Quintile)**															
Lowest (Bottom 20%)	15.9 (15.2-16.7)	8.5 (8.1-9.0)	6.1 (5.7-6.6)	28.8 (27.9-29.8)	18.4 (17.7-19.0)	12.8 (12.3-13.4)	53.6 (52.6-54.6)	67.2 (66.3-68.0)	69.1 (68.3-69.9)	1.6 (1.3-1.8)	5.4 (5.0-5.8)	10.5 (10.0-11.0)	0.1 (0.0-0.2)	0.5 (0.4-0.7)	1.5 (1.3-1.7)
Low	13.9 (13.2-14.5)	7.2 (6.8-7.6)	5.0 (4.6-5.3)	24.6 (23.7-25.4)	13.7 (13.2-14.3)	8.3 (7.9-8.8)	58.9 (58.0-59.9)	67.3 (66.5-68.0)	68.8 (68.1-69.5)	2.4 (2.1-2.7)	10.6 (10.2-11.1)	15.3 (14.8-15.9)	0.3 (0.2-0.4)	1.2 (1.0-1.4)	2.6 (2.4-2.9)
Middle	11.4 (10.9-12.0)	4.8 (4.5-5.1)	3.7 (3.4-4.0)	20.3 (19.6-21.0)	10.0 (9.6-10.4)	7.1 (6.7-7.5)	62.1 (61.2-63.0)	65.6 (64.9-66.2)	63.9 (63.1-64.6)	5.7 (5.3-6.1)	17.0 (16.4-17.5)	21.9 (21.3-22.6)	0.5 (0.4-0.6)	2.7 (2.5-3.0)	3.4 (3.2-3.7)
High	8.4 (7.9-8.9)	3.6 (3.4-3.9)	2.5 (2.3-2.8)	14.5 (13.9-15.1)	6.7 (6.4-7.1)	4.6 (4.3-4.9)	64.6 (63.8-65.4)	61.2 (60.5-61.9)	59.5 (58.8-60.2)	11.2 (10.7-11.8)	23.7 (23.1-24.3)	27.5 (26.9-28.2)	1.3 (1.1-1.5)	4.7 (4.4-5.0)	5.9 (5.5-6.2)
Highest (Top 20%)	4.4 (4.0-4.7)	2.1 (1.9-2.3)	1.8 (1.6-2.0)	7.5 (7.1-7.9)	4.0 (3.7-4.3)	3.4 (3.1-3.7)	59.4 (58.6-60.2)	56.4 (55.7-57.1)	53.2 (52.4-54.0)	23.9 (23.2-24.6)	30.5 (29.8-31.1)	32.8 (32.0-33.5)	4.9 (4.5-5.2)	7.0 (6.7-7.4)	8.8 (8.4-9.3)
**Levels of Schooling**															
No Schooling	14.0 (13.4-14.6)	7.4 (7.0-7.9)	5.4 (5.0-5.9)	25.4 (24.7-26.2)	13.5 (13.0-14.1)	9.1 (8.6-9.7)	56.5 (55.6-57.4)	66.0 (65.2-66.8)	65.9 (65.0-66.8)	3.6 (3.3-4.0)	11.0 (10.5-11.6)	16.8 (16.1-17.5)	0.4 (0.3-0.5)	2.0 (1.8-2.2)	2.8 (2.5-3.1)
1st-5th grade	11.9 (11.3-12.5)	6.0 (5.6-6.4)	4.3 (4.0-4.7)	22.0 (21.2-22.8)	13.1 (12.5-13.7)	9.5 (8.9-10.1)	59.4 (58.5-60.4)	63.1 (62.2-63.9)	66.1 (65.2-67.0)	5.8 (5.4-6.3)	15.3 (14.6-15.9)	16.8 (16.1-17.6)	0.9 (0.8-1.1)	2.6 (2.3-2.9)	3.3 (2.9-3.6)
6th-8th grade	11.5 (10.9-12.1)	5.6 (5.2-6.0)	4.0 (3.7-4.4)	18.6 (17.9-19.4)	11.1 (10.6-11.6)	7.8 (7.4-8.3)	61.0 (60.0-61.9)	64.1 (63.3-64.9)	64.0 (63.1-64.8)	7.6 (7.1-8.2)	16.5 (15.9-17.1)	20.2 (19.5-20.9)	1.3 (1.1-1.5)	2.7 (2.4-2.9)	3.9 (3.6-4.3)
9th-12th grade	8.6 (8.2-9.0)	4.1 (3.9-4.3)	3.4 (3.2-3.6)	15.1 (14.5-15.6)	8.4 (8.1-8.7)	6.0 (5.8-6.3)	61.5 (60.7-62.2)	62.6 (62.0-63.1)	60.9 (60.3-61.5)	12.8 (12.3-13.3)	20.7 (20.3-21.2)	24.2 (23.7-24.7)	2.1 (1.9-2.4)	4.3 (4.0-4.5)	5.5 (5.2-5.8)
>12th grade	5.0 (4.6-5.5)	3.0 (2.8-3.3)	2.4 (2.2-2.7)	8.9 (8.3-9.5)	6.0 (5.6-6.3)	5.2 (4.8-5.5)	62.3 (61.3-63.4)	60.6 (59.9-61.4)	60.4 (59.7-61.2)	20.5 (19.7-21.4)	25.5 (24.8-26.1)	26.6 (26.0-27.3)	3.3 (2.9-3.7)	4.9 (4.6-5.3)	5.3 (5.0-5.7)
**Place of Residence**															
Rural	11.7 (11.3-12.0)	5.6 (5.4-5.8)	4.1 (4.0-4.3)	21.6 (21.1-22.0)	11.7 (11.5-12.0)	8.0 (7.7-8.2)	59.8 (59.3-60.3)	65.5 (65.1-65.9)	65.0 (64.6-65.4)	6.2 (6.0-6.4)	14.7 (14.4-15.0)	19.4 (19.1-19.8)	0.8 (0.7-0.9)	2.4 (2.3-2.6)	3.5 (3.3-3.7)
Urban	7.8 (7.5-8.2)	3.7 (3.5-4.0)	2.9 (2.7-3.1)	12.1 (11.7-12.6)	6.5 (6.2-6.8)	5.1 (4.9-5.4)	60.6 (59.9-61.2)	58.7 (58.2-59.3)	58.0 (57.4-58.6)	16.5 (16.0-17.0)	25.6 (25.1-26.1)	27.3 (26.8-27.9)	3.0 (2.8-3.2)	5.4 (5.2-5.7)	6.6 (6.3-6.9)

Table S3: Relative Change (%) of Body Mass Index (BMI) Prevalence between latest and earliest year, by Highest and Lowest Sociodemographic Characteristics

	**Women**					**Men**				
	Severely/ Moderately Thin	Mildly Thin	Normal	Overweight	Obese	Severely/ Moderately Thin	Mildly Thin	Normal	Overweight	Obese
**Household Wealth (Quintile)**										
Lowest (Bottom 20%)	-58.3	-47.2	34.4	634.3	1149.1	-61.5	-55.1	28.2	554.7	1933.1
Low	-65.6	-57.8	23.6	579.3	1343.7	-64.1	-66.3	16.0	558.7	920.0
Middle	-71.1	-62.7	9.8	334.9	808.3	-67.6	-65.5	2.6	284.1	654.1
High	-69.0	-62.1	-4.9	137.0	396.3	-69.6	-68.5	-7.6	144.7	341.0
Highest (Top 20%)	-52.3	-50.5	-6.6	21.0	70.4	-60.3	-52.8	-10.9	37.8	82.3
**Levels of Schooling**										
No Schooling	-65.3	-57.6	18.6	292.3	477.4	-58.7	-59.7	15.2	322.1	516.6
1st-5th grade	-65.7	-56.2	10.2	118.6	203.4	-63.7	-61.3	11.2	217.5	263.5
6th-8th grade	-57.6	-48.3	2.0	64.0	135.8	-62.2	-59.6	2.1	116.0	179.9
9th-12th grade	-15.3	-19.9	0.3	9.1	30.3	-59.5	-53.8	-4.8	75.7	176.1
>12th grade	-20.1	-12.8	4.9	-3.7	7.6	-53.6	-43.8	-2.5	30.2	60.6
**Place of Residence**										
Rural	-62.1	-55.4	-0.2	39.2	91.9	-63.2	-57.8	-4.1	65.7	119.3
Urban	-63.7	-54.8	14.2	229.8	420.3	-64.4	-62.9	8.3	214.3	352.9

Note: Relative change was calculated as the difference between prevalence at the latest year and the prevalence at the earliest year, divided by prevalence at the earliest year, and shown in percentages. The latest year of prevalence is 2021 for both men and women. The earliest year is 1999 for women and 2006 for men.

Table S4: Age-adjusted and mutually adjusted odds ratios (OR) and 95% Confidence Intervals for indicators of socioeconomic positions for BMI outcomes, Severely / Moderately Thin & Mildly Thin (A), Normal (B), and Overweight & Obesity(C)

A. Severely / Moderately Thin & Mildly Thin

	**Severely / Moderately Thin**							**Mildly Thin**						
	Women				Men			Women				Men		
	1999	2006	2016	2021	1999	2006	2016	1999	2006	2016	2021	2006	2016	2021
**Wealth Quintile**														
Unadjusted														
Lowest (Bottom 20%)	Ref*	Ref	Ref	Ref	Ref	Ref	Ref	Ref	Ref	Ref	Ref	Ref	Ref	Ref
Low	0.88 (0.83-0.93)	0.84 (0.79-0.88)	0.74 (0.72-0.76)	0.72 (0.70-0.75)	0.85 (0.79,0.92)	0.83 (0.77,0.91)	0.80 (0.72,0.89)	0.91 (0.86,0.96)	0.83 (0.79,0.87)	0.74 (0.72,0.76)	0.72 (0.70,0.74)	0.80 (0.75,0.86)	0.71 (0.67,0.75)	0.61 (0.57,0.66)
Middle	0.73 (0.69-0.78)	0.60 (0.56-0.63)	0.50 (0.49-0.52)	0.52 (0.50-0.53)	0.68 (0.63,0.74)	0.54 (0.49,0.59)	0.58 (0.52,0.65)	0.77 (0.72,0.81)	0.65 (0.61,0.68)	0.52 (0.51,0.54)	0.54 (0.52,0.55)	0.63 (0.59,0.67)	0.49 (0.46,0.53)	0.52 (0.48,0.56)
High	0.47 (0.44-0.51)	0.37 (0.35-0.39)	0.34 (0.33-0.35)	0.38 (0.36-0.39)	0.49 (0.45,0.53)	0.41 (0.37,0.45)	0.40 (0.35,0.45)	0.54 (0.51,0.57)	0.43 (0.41,0.45)	0.35 (0.34,0.36)	0.41 (0.39,0.42)	0.42 (0.39,0.45)	0.32 (0.30,0.34)	0.33 (0.30,0.36)
Highest (Top 20%)	0.19 (0.17-0.20)	0.19 (0.18-0.20)	0.20 (0.19-0.20)	0.25 (0.24-0.26)	0.24 (0.22,0.27)	0.23 (0.21,0.26)	0.27 (0.24,0.31)	0.25 (0.23,0.26)	0.23 (0.22,0.25)	0.22 (0.21,0.22)	0.26 (0.25,0.27)	0.20 (0.18,0.22)	0.18 (0.17,0.20)	0.24 (0.22,0.27)
Age-adjusted														
Lowest (Bottom 20%)	Ref	Ref	Ref	Ref	Ref	Ref	Ref	Ref	Ref	Ref	Ref	Ref	Ref	Ref
Low	0.88 (0.83-0.93)	0.84 (0.79-0.88)	0.73 (0.71-0.75)	0.71 (0.69-0.73)	0.84 (0.77,0.91)	0.82 (0.75,0.89)	0.78 (0.70,0.86)	0.92 (0.87,0.97)	0.83 (0.79,0.87)	0.73 (0.71,0.74)	0.70 (0.69,0.72)	0.78 (0.74,0.84)	0.69 (0.65,0.74)	0.60 (0.56,0.65)
Middle	0.73 (0.69-0.78)	0.59 (0.56-0.63)	0.49 (0.47-0.50)	0.51 (0.49-0.52)	0.67 (0.62,0.72)	0.53 (0.49,0.58)	0.57 (0.51,0.64)	0.78 (0.73,0.82)	0.64 (0.61,0.68)	0.51 (0.49,0.52)	0.53 (0.52,0.55)	0.60 (0.57,0.64)	0.48 (0.45,0.51)	0.51 (0.47,0.55)
High	0.47 (0.45-0.51)	0.37 (0.35-0.39)	0.33 (0.32-0.34)	0.37 (0.36-0.38)	0.47 (0.44,0.51)	0.40 (0.36,0.44)	0.39 (0.35,0.44)	0.55 (0.52,0.58)	0.42 (0.40,0.45)	0.33 (0.32,0.34)	0.40 (0.39,0.41)	0.40 (0.37,0.43)	0.31 (0.29,0.34)	0.32 (0.30,0.35)
Highest (Top 20%)	0.19 (0.17-0.21)	0.19 (0.18-0.20)	0.19 (0.19-0.20)	0.25 (0.24-0.26)	0.24 (0.21,0.26)	0.23 (0.21,0.26)	0.27 (0.23,0.31)	0.26 (0.24,0.27)	0.23 (0.22,0.25)	0.21 (0.21,0.22)	0.26 (0.25,0.27)	0.19 (0.18,0.21)	0.18 (0.17,0.20)	0.24 (0.22,0.27)
Multivariate														
Lowest (Bottom 20%)	Ref	Ref	Ref	Ref	Ref	Ref	Ref	Ref	Ref	Ref	Ref	Ref	Ref	Ref
Low	0.89 (0.83-0.94)	0.86 (0.81-0.90)	0.77 (0.75-0.79)	0.75 (0.72-0.77)	0.85 (0.79,0.92)	0.87 (0.80,0.95)	0.83 (0.75,0.93)	0.94 (0.89,1.00)	0.86 (0.82,0.91)	0.78 (0.76,0.80)	0.75 (0.73,0.77)	0.84 (0.78,0.89)	0.73 (0.69,0.78)	0.64 (0.59,0.69)
Middle	0.75 (0.71-0.80)	0.62 (0.59-0.66)	0.55 (0.53-0.57)	0.56 (0.54-0.58)	0.68 (0.63,0.74)	0.58 (0.53,0.64)	0.64 (0.57,0.72)	0.83 (0.79,0.88)	0.70 (0.66,0.74)	0.59 (0.57,0.60)	0.60 (0.58,0.61)	0.69 (0.64,0.73)	0.53 (0.50,0.57)	0.57 (0.53,0.62)
High	0.51 (0.47-0.54)	0.40 (0.37-0.43)	0.40 (0.38-0.41)	0.43 (0.41-0.44)	0.49 (0.45,0.54)	0.45 (0.40,0.50)	0.46 (0.40,0.53)	0.64 (0.60,0.68)	0.51 (0.48,0.54)	0.43 (0.41,0.44)	0.48 (0.46,0.50)	0.50 (0.47,0.54)	0.37 (0.35,0.41)	0.38 (0.34,0.42)
Highest (Top 20%)	0.22 (0.20-0.25)	0.21 (0.19-0.23)	0.25 (0.24-0.26)	0.29 (0.28-0.31)	0.26 (0.23,0.30)	0.27 (0.24,0.31)	0.34 (0.29,0.41)	0.36 (0.33,0.40)	0.31 (0.29,0.34)	0.29 (0.28,0.31)	0.34 (0.32,0.35)	0.29 (0.26,0.32)	0.24 (0.22,0.27)	0.30 (0.26,0.34)
**Levels of Schooling**														
Unadjusted														
No Schooling	Ref	Ref	Ref	Ref	Ref	Ref	Ref	Ref	Ref	Ref	Ref	Ref	Ref	Ref
1st-5th grade	0.76 (0.72-0.80)	0.70 (0.66-0.74)	0.81 (0.79-0.84)	0.78 (0.75-0.81)	0.83 (0.76,0.89)	0.79 (0.72,0.87)	0.79 (0.69,0.89)	0.78 (0.74,0.82)	0.75 (0.72,0.79)	0.80 (0.78,0.82)	0.83 (0.81,0.86)	0.83 (0.78,0.88)	0.96 (0.89,1.03)	1.04 (0.95,1.15)
6th-8th grade	0.60 (0.56-0.64)	0.64 (0.60-0.68)	0.71 (0.69-0.74)	0.77 (0.74-0.80)	0.79 (0.73,0.86)	0.74 (0.67,0.81)	0.73 (0.65,0.83)	0.60 (0.57,0.64)	0.68 (0.64,0.71)	0.71 (0.69,0.73)	0.82 (0.79,0.84)	0.67 (0.63,0.72)	0.80 (0.74,0.85)	0.85 (0.77,0.93)
9th-12th grade	0.41 (0.38-0.44)	0.48 (0.45-0.50)	0.60 (0.58-0.61)	0.78 (0.75-0.80)	0.57 (0.53,0.62)	0.53 (0.49,0.58)	0.61 (0.55,0.68)	0.45 (0.42,0.48)	0.54 (0.51,0.57)	0.61 (0.60,0.63)	0.79 (0.77,0.81)	0.52 (0.49,0.55)	0.58 (0.55,0.62)	0.64 (0.59,0.69)
>12th grade	0.22 (0.18-0.26)	0.36 (0.33-0.40)	0.54 (0.52-0.56)	0.73 (0.71-0.76)	0.33 (0.29,0.36)	0.39 (0.35,0.44)	0.43 (0.38,0.49)	0.26 (0.23,0.30)	0.38 (0.35,0.42)	0.56 (0.54,0.58)	0.75 (0.73,0.77)	0.29 (0.26,0.31)	0.40 (0.37,0.44)	0.54 (0.49,0.60)
Age-adjusted														
No Schooling	Ref	Ref	Ref	Ref	Ref	Ref	Ref	Ref	Ref	Ref	Ref	Ref	Ref	Ref
1st-5th grade	0.76 (0.72-0.80)	0.67 (0.63-0.71)	0.70 (0.68-0.73)	0.67 (0.65-0.70)	0.81 (0.75,0.87)	0.76 (0.68,0.84)	0.73 (0.64,0.83)	0.77 (0.73,0.81)	0.71 (0.68,0.75)	0.68 (0.66,0.70)	0.71 (0.69,0.74)	0.79 (0.75,0.85)	0.91 (0.84,0.98)	0.96 (0.87,1.05)
6th-8th grade	0.58 (0.54-0.62)	0.58 (0.55-0.62)	0.56 (0.54-0.58)	0.57 (0.55-0.59)	0.75 (0.70,0.82)	0.66 (0.60,0.73)	0.63 (0.55,0.71)	0.56 (0.53,0.60)	0.59 (0.56,0.63)	0.54 (0.52,0.55)	0.60 (0.59,0.62)	0.60 (0.56,0.64)	0.69 (0.64,0.74)	0.71 (0.65,0.78)
9th-12th grade	0.39 (0.36-0.42)	0.42 (0.40-0.45)	0.44 (0.42-0.45)	0.50 (0.48-0.51)	0.55 (0.51,0.59)	0.47 (0.43,0.51)	0.48 (0.43,0.53)	0.41 (0.39,0.44)	0.45 (0.43,0.48)	0.43 (0.42,0.44)	0.51 (0.49,0.52)	0.46 (0.43,0.49)	0.49 (0.46,0.53)	0.50 (0.46,0.55)
>12th grade	0.21 (0.18-0.25)	0.32 (0.29-0.35)	0.34 (0.33-0.36)	0.40 (0.39-0.42)	0.31 (0.28,0.35)	0.31 (0.28,0.35)	0.29 (0.25,0.33)	0.25 (0.22,0.29)	0.31 (0.29,0.34)	0.34 (0.33,0.35)	0.41 (0.40,0.43)	0.25 (0.23,0.27)	0.30 (0.28,0.33)	0.37 (0.34,0.41)
Multivariate														
No Schooling	Ref	Ref	Ref	Ref	Ref	Ref	Ref	Ref	Ref	Ref	Ref	Ref	Ref	Ref
1st-5th grade	0.96 (0.90-1.02)	0.85 (0.80-0.90)	0.85 (0.83-0.88)	0.77 (0.74-0.80)	0.92 (0.85,0.99)	0.84 (0.76,0.93)	0.77 (0.68,0.88)	0.92 (0.87,0.97)	0.87 (0.82,0.92)	0.81 (0.79,0.84)	0.81 (0.79,0.84)	0.91 (0.86,0.97)	1.04 (0.97,1.12)	1.04 (0.94,1.14)

6th-8th grade	0.93 (0.86-1.00)	0.92 (0.86-0.98)	0.80 (0.77-0.82)	0.74 (0.71-0.76)	0.98 (0.90,1.06)	0.82 (0.75,0.91)	0.72 (0.63,0.81)	0.81 (0.75,0.86)	0.87 (0.82,0.92)	0.75 (0.73,0.77)	0.77 (0.75,0.79)	0.80 (0.74,0.85)	0.91 (0.84,0.98)	0.86 (0.78,0.94)
9th-12th grade	0.83 (0.77-0.91)	0.90 (0.84-0.96)	0.76 (0.74-0.79)	0.76 (0.74-0.79)	0.86 (0.79,0.93)	0.69 (0.63,0.76)	0.65 (0.58,0.73)	0.74 (0.68,0.79)	0.84 (0.79,0.89)	0.73 (0.71,0.75)	0.76 (0.73,0.78)	0.74 (0.69,0.79)	0.79 (0.74,0.85)	0.73 (0.67,0.80)
>12th grade	0.68 (0.57-0.82)	1.00 (0.90-1.11)	0.84 (0.80-0.88)	0.81 (0.77-0.85)	0.65 (0.58,0.74)	0.59 (0.52,0.67)	0.48 (0.41,0.55)	0.61 (0.53,0.71)	0.81 (0.73,0.89)	0.77 (0.74,0.80)	0.80 (0.77,0.83)	0.55 (0.50,0.61)	0.64 (0.59,0.71)	0.66 (0.59,0.74)
**Residence**														
Unadjusted														
Rural	Ref	Ref	Ref	Ref	Ref	Ref	Ref	Ref	Ref	Ref	Ref	Ref	Ref	Ref
Urban	0.48 (0.45-0.50)	0.49 (0.47-0.52)	0.47 (0.46-0.48)	0.53 (0.52-0.55)	0.64 (0.61,0.68)	0.65 (0.61,0.70)	0.69 (0.64,0.76)	0.47 (0.45,0.50)	0.47 (0.45,0.49)	0.46 (0.45,0.47)	0.50 (0.49,0.52)	0.50 (0.48,0.53)	0.52 (0.50,0.55)	0.62 (0.59,0.67)
Age-adjusted														
Rural	Ref	Ref	Ref	Ref	Ref	Ref	Ref	Ref	Ref	Ref	Ref	Ref	Ref	Ref
Urban	0.48 (0.46,0.51)	0.49 (0.47,0.52)	0.47 (0.46,0.48)	0.54 (0.53,0.56)	0.64 (0.60,0.68)	0.65 (0.61,0.70)	0.69 (0.64,0.75)	0.48 (0.46,0.51)	0.47 (0.45,0.48)	0.46 (0.45,0.47)	0.51 (0.50,0.52)	0.49 (0.47,0.52)	0.52 (0.50,0.55)	0.62 (0.58,0.66)
Multivariate														
Rural	Ref	Ref	Ref	Ref	Ref	Ref	Ref	Ref	Ref	Ref	Ref	Ref	Ref	Ref
Urban	0.97 (0.91,1.03)	0.95 (0.90,1.00)	0.83 (0.81,0.86)	0.88 (0.85,0.91)	1.13 (1.05,1.21)	1.12 (1.03,1.21)	1.10 (1.00,1.22)	0.84 (0.80,0.89)	0.78 (0.75,0.82)	0.78 (0.76,0.80)	0.78 (0.76,0.80)	0.87 (0.82,0.92)	0.91 (0.86,0.97)	1.00 (0.92,1.07)

Note: *Ref refers to the reference category. p-value was smaller than 0.0001 for all models**.** The cutoff points of BMI categories are: Severely/Moderately Thin (<17.0), Mildly Thin (16.0-18.4), Normal (18.5-24.9), Overweight (25.0-29.9), and Obese (≥30.0).

B. Normal

	Women				Men		
	1999	2006	2016	2021	2006	2016	2021
**Wealth Quintile**							
Unadjusted							
Lowest (Bottom 20%)	Ref*	Ref	Ref	Ref	Ref	Ref	Ref
Low	1.13 (1.08,1.19)	1.18 (1.13,1.23)	1.03 (1.02,1.05)	0.94 (0.93,0.96)	1.24 (1.17,1.31)	1.00 (0.96,1.05)	0.99 (0.94,1.04)
Middle	1.28 (1.22,1.34)	1.44 (1.38,1.50)	0.98 (0.96,1.00)	0.81 (0.80,0.83)	1.42 (1.34,1.50)	0.93 (0.89,0.98)	0.79 (0.75,0.83)
High	1.52 (1.45,1.60)	1.52 (1.46,1.59)	0.82 (0.80,0.83)	0.70 (0.69,0.71)	1.58 (1.50,1.67)	0.77 (0.74,0.81)	0.66 (0.63,0.69)
Highest (Top 20%)	1.31 (1.25,1.37)	1.19 (1.14,1.25)	0.72 (0.70,0.73)	0.58 (0.57,0.59)	1.27 (1.20,1.34)	0.63 (0.60,0.66)	0.51 (0.49,0.54)
Age-adjusted							
Lowest (Bottom 20%)	Ref	Ref	Ref	Ref	Ref	Ref	Ref
Low	1.13 (1.08,1.19)	1.18 (1.13,1.23)	1.03 (1.01,1.05)	0.94 (0.92,0.96)	1.25 (1.18,1.32)	1.00 (0.95,1.05)	0.98 (0.93,1.03)
Middle	1.28 (1.22,1.35)	1.44 (1.38,1.51)	0.97 (0.96,0.99)	0.81 (0.80,0.83)	1.42 (1.35,1.51)	0.92 (0.88,0.96)	0.79 (0.75,0.83)
High	1.53 (1.46,1.61)	1.53 (1.46,1.59)	0.81 (0.80,0.83)	0.70 (0.69,0.71)	1.58 (1.50,1.67)	0.77 (0.73,0.80)	0.66 (0.63,0.69)
Highest (Top 20%)	1.32 (1.26,1.38)	1.20 (1.15,1.25)	0.72 (0.70,0.73)	0.58 (0.57,0.59)	1.27 (1.21,1.34)	0.63 (0.60,0.66)	0.51 (0.48,0.54)
Multivariate							
Lowest (Bottom 20%)	Ref	Ref	Ref	Ref	Ref	Ref	Ref
Low	1.14 (1.08,1.19)	1.20 (1.14,1.25)	1.06 (1.04,1.08)	0.97 (0.96,0.99)	1.23 (1.16,1.30)	1.02 (0.97,1.07)	1.00 (0.95,1.05)
Middle	1.30 (1.24,1.37)	1.49 (1.43,1.56)	1.03 (1.01,1.05)	0.86 (0.85,0.88)	1.39 (1.31,1.48)	0.96 (0.92,1.01)	0.82 (0.78,0.86)
High	1.58 (1.50,1.67)	1.64 (1.56,1.72)	0.89 (0.87,0.91)	0.76 (0.75,0.78)	1.54 (1.44,1.64)	0.83 (0.79,0.88)	0.69 (0.66,0.73)
Highest (Top 20%)	1.39 (1.31,1.48)	1.32 (1.25,1.40)	0.78 (0.77,0.80)	0.64 (0.63,0.66)	1.22 (1.13,1.31)	0.72 (0.68,0.76)	0.54 (0.51,0.58)
**Levels of Schooling**							
Unadjusted							
No Schooling	Ref	Ref	Ref	Ref	Ref	Ref	Ref
1st-5th grade	1.06 (1.02,1.10)	1.10 (1.06,1.15)	0.92 (0.90,0.93)	0.89 (0.88,0.91)	1.13 (1.07,1.19)	0.88 (0.84,0.93)	1.01 (0.95,1.07)
6th-8th grade	1.13 (1.08,1.18)	1.02 (0.98,1.06)	0.89 (0.87,0.90)	0.83 (0.81,0.84)	1.20 (1.14,1.27)	0.92 (0.88,0.97)	0.92 (0.87,0.97)
9th-12th grade	1.19 (1.14,1.25)	1.06 (1.03,1.10)	0.87 (0.86,0.88)	0.79 (0.78,0.81)	1.23 (1.17,1.29)	0.86 (0.83,0.90)	0.81 (0.77,0.84)

>12th grade	1.10 (1.02,1.19)	1.11 (1.05,1.16)	0.98 (0.97,1.00)	0.87 (0.85,0.88)	1.27 (1.20,1.35)	0.79 (0.76,0.83)	0.79 (0.75,0.83)
Age-adjusted							
No Schooling	Ref	Ref	Ref	Ref	Ref	Ref	Ref
1st-5th grade	1.06 (1.01,1.10)	1.09 (1.05,1.14)	0.87 (0.85,0.88)	0.84 (0.83,0.86)	1.13 (1.07,1.19)	0.85 (0.81,0.90)	0.98 (0.92,1.03)
6th-8th grade	1.12 (1.07,1.18)	1.00 (0.96,1.04)	0.81 (0.80,0.83)	0.74 (0.73,0.75)	1.20 (1.14,1.27)	0.85 (0.81,0.89)	0.86 (0.82,0.91)
9th-12th grade	1.18 (1.13,1.24)	1.04 (1.00,1.07)	0.78 (0.77,0.79)	0.68 (0.67,0.69)	1.22 (1.16,1.28)	0.79 (0.76,0.82)	0.74 (0.71,0.78)
>12th grade	1.09 (1.02,1.18)	1.07 (1.02,1.13)	0.85 (0.83,0.86)	0.70 (0.69,0.71)	1.26 (1.19,1.34)	0.70 (0.67,0.73)	0.69 (0.65,0.72)
Multivariate	0.00	0.00	0.00	0.00			
No Schooling	Ref	Ref	Ref	Ref	Ref	Ref	Ref
1st-5th grade	0.97 (0.93,1.01)	1.01 (0.97,1.05)	0.91 (0.89,0.93)	0.89 (0.88,0.91)	1.06 (1.00,1.12)	0.88 (0.84,0.93)	1.02 (0.96,1.08)
6th-8th grade	0.99 (0.94,1.05)	0.90 (0.86,0.94)	0.89 (0.87,0.90)	0.83 (0.81,0.84)	1.09 (1.03,1.16)	0.92 (0.87,0.97)	0.95 (0.90,1.00)
9th-12th grade	1.04 (0.99,1.10)	0.94 (0.90,0.98)	0.91 (0.90,0.93)	0.82 (0.81,0.84)	1.10 (1.04,1.16)	0.91 (0.87,0.96)	0.91 (0.86,0.96)
>12th grade	0.99 (0.91,1.08)	1.03 (0.97,1.10)	1.10 (1.08,1.12)	0.95 (0.93,0.97)	1.18 (1.10,1.27)	0.90 (0.85,0.95)	0.97 (0.92,1.03)
**Residence**							
Unadjusted							
Rural	Ref	Ref	Ref	Ref	Ref	Ref	Ref
Urban	1.04 (1.01,1.08)	0.98 (0.95,1.00)	0.77 (0.76,0.78)	0.77 (0.76,0.77)	1.03 (1.00,1.07)	0.75 (0.73,0.77)	0.74 (0.72,0.77)
Age-adjusted							
Rural	Ref	Ref	Ref	Ref	Ref	Ref	Ref
Urban	1.04 (1.01,1.08)	0.98 (0.95,1.00)	0.77 (0.76,0.78)	0.77 (0.76,0.78)	1.03 (1.00,1.07)	0.75 (0.73,0.77)	0.74 (0.72,0.77)
Multivariate							
Rural	Ref	Ref	Ref	Ref	Ref	Ref	Ref
Urban	0.91 (0.87,0.94)	0.90 (0.87,0.93)	0.86 (0.85,0.87)	0.93 (0.92,0.95)	0.95 (0.91,0.99)	0.87 (0.84,0.90)	0.95 (0.91,0.98)

Note: *Ref refers to the reference category. p-value was smaller than 0.0001 for all models**.** The cutoff points of BMI categories are: Severely/Moderately Thin (<17.0), Mildly Thin (16.0-18.4), Normal (18.5-24.9), Overweight (25.0-29.9), and Obese (≥30.0).

C. Overweight & Obesity

	Overweight							Obese						
	Women				Men			Women				Men		
	1999	2006	2016	2021	1999	2006	2016	1999	2006	2016	2021	2006	2016	2021
**Wealth Quintile**														
Unadjusted														
Lowest (Bottom 20%)	Ref*	Ref	Ref	Ref	Ref	Ref	Ref	Ref	Ref	Ref	Ref	Ref	Ref	Ref
Low	1.70 (1.42,2.05)	2.39 (2.07,2.76)	1.99 (1.93,2.06)	1.67 (1.62,1.71)	1.53 (1.25,1.88)	2.07 (1.89,2.27)	1.55 (1.44,1.66)	1.69 (0.98,2.94)	2.02 (1.43,2.86)	2.33 (2.15,2.52)	1.99 (1.89,2.11)	3.50 (1.55,7.94)	2.19 (1.66,2.87)	1.78 (1.49,2.12)
Middle	3.63 (3.07,4.28)	4.80 (4.20,5.49)	3.29 (3.19,3.40)	2.36 (2.30,2.42)	3.78 (3.16,4.53)	3.57 (3.28,3.88)	2.40 (2.25,2.57)	4.74 (2.94,7.64)	3.84 (2.80,5.28)	4.88 (4.54,5.25)	3.60 (3.42,3.79)	6.04 (2.78,13.13)	5.10 (3.98,6.55)	2.36 (2.00,2.78)
High	8.34 (7.13,9.76)	10.00 (8.80,11.36)	4.81 (4.67,4.96)	2.92 (2.85,3.00)	7.89 (6.64,9.37)	5.43 (5.00,5.90)	3.25 (3.04,3.47)	13.10 (8.34,20.58)	12.01 (8.92,16.17)	9.62 (8.97,10.32)	5.56 (5.29,5.84)	17.21 (8.17,36.28)	9.04 (7.09,11.52)	4.12 (3.52,4.81)
Highest (Top 20%)	22.30 (19.16,25.96)	19.28 (17.01,21.86)	6.03 (5.85,6.22)	3.58 (3.50,3.67)	19.61 (16.58,23.19)	7.65 (7.05,8.30)	4.17 (3.90,4.45)	57.89 (37.30,89.86)	36.26 (27.10,48.51)	14.49 (13.52,15.53)	8.28 (7.88,8.69)	66.10 (31.72,137.76)	13.81 (10.86,17.55)	6.40 (5.49,7.46)
Age-adjusted														
Lowest (Bottom 20%)	Ref	Ref	Ref	Ref	Ref	Ref	Ref	Ref	Ref	Ref	Ref	Ref	Ref	Ref
Low	1.68 (1.39,2.02)	2.38 (2.06,2.75)	2.04 (1.97,2.11)	1.70 (1.65,1.74)	1.59 (1.29,1.95)	2.14 (1.95,2.34)	1.58 (1.47,1.69)	1.66 (0.96,2.87)	1.99 (1.41,2.82)	2.37 (2.19,2.56)	2.02 (1.91,2.14)	3.63 (1.60,8.22)	2.24 (1.71,2.95)	1.80 (1.51,2.14)
Middle	3.52 (2.98,4.16)	4.85 (4.25,5.55)	3.42 (3.32,3.53)	2.40 (2.34,2.46)	4.03 (3.36,4.83)	3.72 (3.41,4.05)	2.45 (2.29,2.62)	4.54 (2.81,7.32)	3.81 (2.77,5.24)	5.03 (4.68,5.41)	3.63 (3.45,3.83)	6.42 (2.95,13.95)	5.27 (4.11,6.77)	2.38 (2.02,2.80)
High	8.20 (7.01,9.59)	10.36 (9.11,11.77)	5.06 (4.91,5.22)	3.00 (2.92,3.07)	8.57 (7.21,10.18)	5.64 (5.19,6.13)	3.30 (3.09,3.52)	12.56 (8.00,19.73)	12.16 (9.03,16.38)	10.00 (9.32,10.72)	5.66 (5.38,5.95)	18.53 (8.79,39.06)	9.23 (7.24,11.76)	4.13 (3.53,4.83)

Highest (Top 20%)	21.01 (18.04,24.46)	19.83 (17.48,22.48)	6.24 (6.06,6.44)	3.63 (3.54,3.72)	20.90 (17.67,24.73)	7.89 (7.27,8.57)	4.25 (3.98,4.55)	52.11 (33.56,80.91)	36.02 (26.91,48.20)	14.78 (13.79,15.84)	8.31 (7.92,8.73)	68.54 (32.88,142.85)	13.93 (10.96,17.71)	6.44 (5.52,7.51)
Multivariate														
Lowest (Bottom 20%)	Ref	Ref	Ref	Ref	Ref	Ref	Ref	Ref	Ref	Ref	Ref	Ref	Ref	Ref
Low	1.57 (1.30,1.89)	2.20 (1.90,2.54)	1.91 (1.85,1.98)	1.62 (1.57,1.66)	1.46 (1.19,1.80)	1.98 (1.81,2.17)	1.50 (1.40,1.61)	1.54 (0.89,2.67)	1.81 (1.28,2.56)	2.18 (2.01,2.36)	1.87 (1.77,1.98)	3.38 (1.49,7.68)	2.16 (1.64,2.84)	1.71 (1.44,2.04)
Middle	3.01 (2.54,3.56)	4.09 (3.57,4.68)	2.99 (2.89,3.08)	2.19 (2.13,2.25)	3.41 (2.84,4.11)	3.24 (2.97,3.53)	2.25 (2.09,2.41)	3.77 (2.33,6.09)	3.04 (2.20,4.19)	4.15 (3.85,4.46)	3.11 (2.96,3.28)	5.60 (2.56,12.26)	4.86 (3.77,6.26)	2.18 (1.84,2.58)
High	5.96 (5.06,7.01)	7.36 (6.44,8.41)	4.03 (3.90,4.17)	2.61 (2.54,2.69)	6.37 (5.31,7.64)	4.53 (4.15,4.94)	2.90 (2.70,3.11)	8.41 (5.31,13.32)	7.70 (5.67,10.45)	7.14 (6.64,7.68)	4.44 (4.21,4.68)	14.93 (6.98,31.97)	7.96 (6.19,10.23)	3.62 (3.07,4.28)
Highest (Top 20%)	11.51 (9.73,13.62)	11.23 (9.78,12.90)	4.62 (4.46,4.78)	3.03 (2.95,3.13)	12.94 (10.73,15.61)	5.65 (5.16,6.20)	3.53 (3.27,3.82)	24.76 (15.62,39.26)	18.57 (13.63,25.29)	9.62 (8.92,10.37)	6.10 (5.78,6.44)	51.77 (24.16,110.93)	11.14 (8.62,14.39)	5.54 (4.66,6.58)
**Levels of Schooling**														
Unadjusted														
No Schooling	Ref	Ref	Ref	Ref	Ref	Ref	Ref	Ref	Ref	Ref	Ref	Ref	Ref	Ref
1st-5th grade	2.31 (2.13,2.50)	1.91 (1.79,2.04)	1.39 (1.36,1.42)	1.26 (1.23,1.29)	1.63 (1.44,1.85)	1.45 (1.35,1.56)	1.00 (0.93,1.08)	2.76 (2.35,3.24)	2.21 (1.95,2.50)	1.55 (1.49,1.61)	1.44 (1.39,1.50)	2.31 (1.64,3.27)	1.32 (1.12,1.55)	1.18 (1.01,1.38)
6th-8th grade	3.35 (3.09,3.63)	2.43 (2.28,2.59)	1.56 (1.53,1.59)	1.34 (1.31,1.37)	2.18 (1.93,2.46)	1.59 (1.49,1.71)	1.26 (1.18,1.35)	4.43 (3.79,5.18)	3.41 (3.04,3.82)	1.88 (1.81,1.95)	1.70 (1.64,1.75)	3.25 (2.33,4.52)	1.36 (1.16,1.59)	1.42 (1.23,1.65)
9th-12th grade	4.68 (4.35,5.02)	3.18 (3.01,3.36)	1.74 (1.71,1.77)	1.39 (1.37,1.42)	3.87 (3.49,4.30)	2.11 (1.98,2.24)	1.59 (1.50,1.68)	6.29 (5.48,7.23)	4.27 (3.86,4.73)	2.17 (2.10,2.23)	1.82 (1.77,1.87)	5.40 (4.01,7.28)	2.19 (1.92,2.50)	2.02 (1.78,2.30)
>12th grade	7.32 (6.65,8.06)	4.19 (3.91,4.48)	1.64 (1.60,1.68)	1.33 (1.31,1.36)	6.82 (6.11,7.60)	2.75 (2.58,2.93)	1.80 (1.70,1.92)	10.46 (8.84,12.37)	4.44 (3.92,5.02)	1.94 (1.87,2.01)	1.64 (1.59,1.70)	8.38 (6.17,11.37)	2.56 (2.23,2.94)	1.97 (1.72,2.25)
Age-adjusted														
No Schooling	Ref	Ref	Ref	Ref	Ref	Ref	Ref	Ref	Ref	Ref	Ref	Ref	Ref	Ref
1st-5th grade	2.40 (2.21,2.60)	2.10 (1.96,2.25)	1.62 (1.58,1.66)	1.41 (1.38,1.44)	1.73 (1.52,1.96)	1.56 (1.45,1.68)	1.07 (1.00,1.15)	2.86 (2.43,3.36)	2.43 (2.14,2.76)	1.84 (1.77,1.91)	1.66 (1.60,1.72)	2.45 (1.73,3.46)	1.41 (1.20,1.67)	1.24 (1.06,1.46)
6th-8th grade	3.97 (3.65,4.31)	3.12 (2.92,3.33)	2.04 (2.00,2.09)	1.68 (1.64,1.72)	2.63 (2.33,2.97)	1.90 (1.77,2.03)	1.44 (1.35,1.54)	5.30 (4.53,6.21)	4.54 (4.04,5.10)	2.57 (2.48,2.67)	2.25 (2.17,2.33)	3.95 (2.83,5.50)	1.62 (1.39,1.90)	1.59 (1.37,1.84)
9th-12th grade	5.87 (5.45,6.32)	4.51 (4.25,4.78)	2.49 (2.44,2.54)	1.96 (1.93,2.00)	4.80 (4.32,5.33)	2.58 (2.43,2.75)	1.92 (1.82,2.04)	8.01 (6.96,9.22)	6.36 (5.73,7.06)	3.27 (3.17,3.38)	2.75 (2.67,2.84)	6.72 (4.98,9.07)	2.68 (2.35,3.07)	2.36 (2.07,2.67)
>12th grade	8.46 (7.66,9.35)	6.42 (5.98,6.89)	2.86 (2.79,2.93)	2.24 (2.19,2.29)	8.68 (7.77,9.69)	3.88 (3.63,4.15)	2.52 (2.37,2.69)	12.10 (10.21,14.35)	7.12 (6.27,8.08)	3.68 (3.54,3.83)	3.09 (2.98,3.20)	10.52 (7.74,14.30)	3.56 (3.09,4.10)	2.57 (2.24,2.95)
Multivariate														
No Schooling	Ref	Ref	Ref	Ref	Ref	Ref	Ref	Ref	Ref	Ref	Ref	Ref	Ref	Ref
1st-5th grade	1.39 (1.28,1.51)	1.32 (1.23,1.41)	1.24 (1.21,1.27)	1.21 (1.18,1.24)	1.13 (1.00,1.29)	1.26 (1.17,1.36)	0.96 (0.89,1.04)	1.37 (1.16,1.62)	1.29 (1.13,1.47)	1.24 (1.19,1.29)	1.29 (1.24,1.34)	1.37 (0.96,1.94)	1.05 (0.89,1.24)	1.08 (0.92,1.27)
6th-8th grade	1.61 (1.47,1.76)	1.45 (1.35,1.56)	1.31 (1.28,1.34)	1.28 (1.25,1.31)	1.22 (1.07,1.38)	1.29 (1.20,1.39)	1.15 (1.07,1.23)	1.60 (1.35,1.90)	1.61 (1.42,1.83)	1.34 (1.29,1.39)	1.43 (1.38,1.49)	1.34 (0.95,1.90)	0.97 (0.82,1.13)	1.18 (1.01,1.37)
9th-12th grade	1.73 (1.59,1.89)	1.57 (1.47,1.68)	1.33 (1.30,1.36)	1.31 (1.28,1.33)	1.56 (1.39,1.75)	1.43 (1.34,1.52)	1.27 (1.19,1.35)	1.62 (1.38,1.90)	1.49 (1.32,1.68)	1.29 (1.24,1.34)	1.38 (1.34,1.43)	1.38 (1.00,1.90)	1.21 (1.05,1.40)	1.34 (1.17,1.53)
>12th grade	1.89 (1.69,2.12)	1.68 (1.54,1.83)	1.24 (1.20,1.27)	1.26 (1.23,1.29)	1.89 (1.66,2.14)	1.68 (1.56,1.80)	1.38 (1.29,1.49)	1.76 (1.45,2.13)	1.13 (0.98,1.31)	1.04 (1.00,1.09)	1.14 (1.10,1.19)	1.30 (0.93,1.82)	1.17 (1.01,1.37)	1.08 (0.93,1.26)
**Residence**														
Unadjusted														
Rural	Ref	Ref	Ref	Ref	Ref	Ref	Ref	Ref	Ref	Ref	Ref	Ref	Ref	Ref
Urban	3.96 (3.76,4.18)	3.18 (3.05,3.31)	2.04 (2.01,2.07)	1.59 (1.57,1.62)	2.99 (2.83,3.17)	2.00 (1.93,2.07)	1.56 (1.51,1.62)	6.35 (5.72,7.05)	5.03 (4.65,5.43)	3.11 (3.04,3.18)	2.38 (2.34,2.43)	3.84 (3.34,4.42)	2.29 (2.13,2.46)	1.95 (1.82,2.09)
Age-adjusted														
Rural	Ref	Ref	Ref	Ref	Ref	Ref	Ref	Ref	Ref	Ref	Ref	Ref	Ref	Ref
Urban	3.84 (3.64,4.05)	3.25 (3.11,3.39)	2.06 (2.03,2.09)	1.58 (1.56,1.60)	3.11 (2.94,3.29)	2.02 (1.95,2.09)	1.57 (1.52,1.63)	6.02 (5.42,6.69)	5.10 (4.71,5.51)	3.13 (3.06,3.20)	2.36 (2.31,2.41)	3.94 (3.43,4.54)	2.30 (2.14,2.47)	1.96 (1.83,2.09)
Multivariate														
Rural	Ref	Ref	Ref	Ref	Ref	Ref	Ref	Ref	Ref	Ref	Ref	Ref	Ref	Ref
Urban	1.33 (1.25,1.42)	1.38 (1.31,1.45)	1.23 (1.21,1.25)	1.07 (1.06,1.09)	1.18 (1.10,1.26)	1.17 (1.13,1.22)	1.01 (0.97,1.06)	1.68 (1.49,1.90)	1.80 (1.65,1.97)	1.58 (1.54,1.63)	1.34 (1.30,1.37)	1.22 (1.04,1.42)	1.19 (1.10,1.29)	1.14 (1.05,1.23)

Note: *Ref refers to the reference category. p-value was smaller than 0.0001 for all models**.** The cutoff points of BMI categories are: Severely/Moderately Thin (<17.0), Mildly Thin (16.0-18.4), Normal (18.5-24.9), Overweight (25.0-29.9), and Obese (≥30.0).

Figure S1: Trend of weighted Prevalence of Body Mass Index (BMI) categories by age group and residence, Women (A) and Men (B)

A. Women

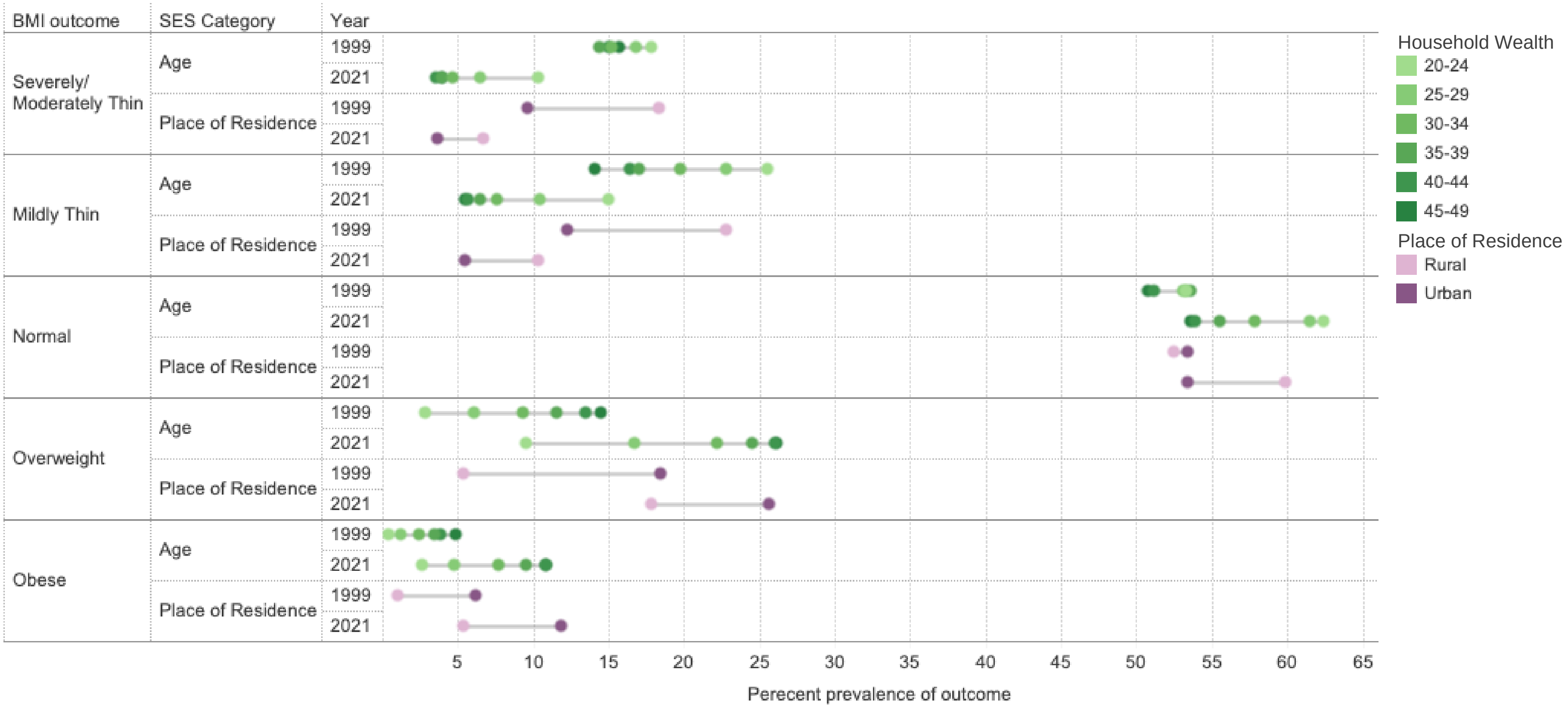

B. Men

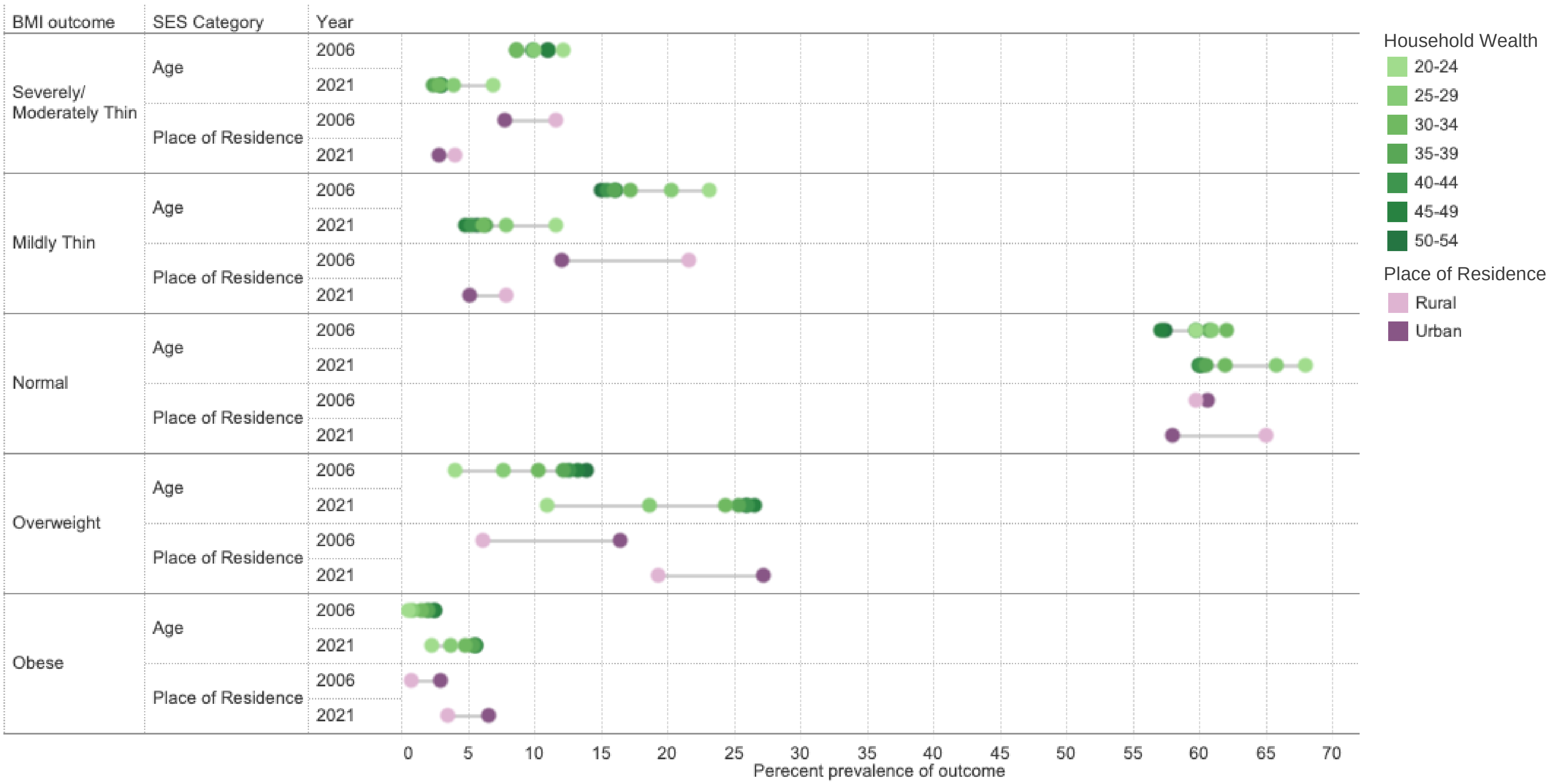

BMI outcome
SES Category
Year
Severely/ Moderately Thin
Mildly Thin
Normal
Overweight
Obese
Age
Place of Residence
2006
2021
0
5
10
15
20
25
30
35
40
45
50
55
60
65
70
Perecent prevalence of outcome
Household Wealth
20-24
25-29
30-34
35-39
40-44
45-49
50-54
Place of Residence
Rural
Urban

Figure S2: Difference in Percent Prevalence of the lowest and highest SES groups by BMI outcomes

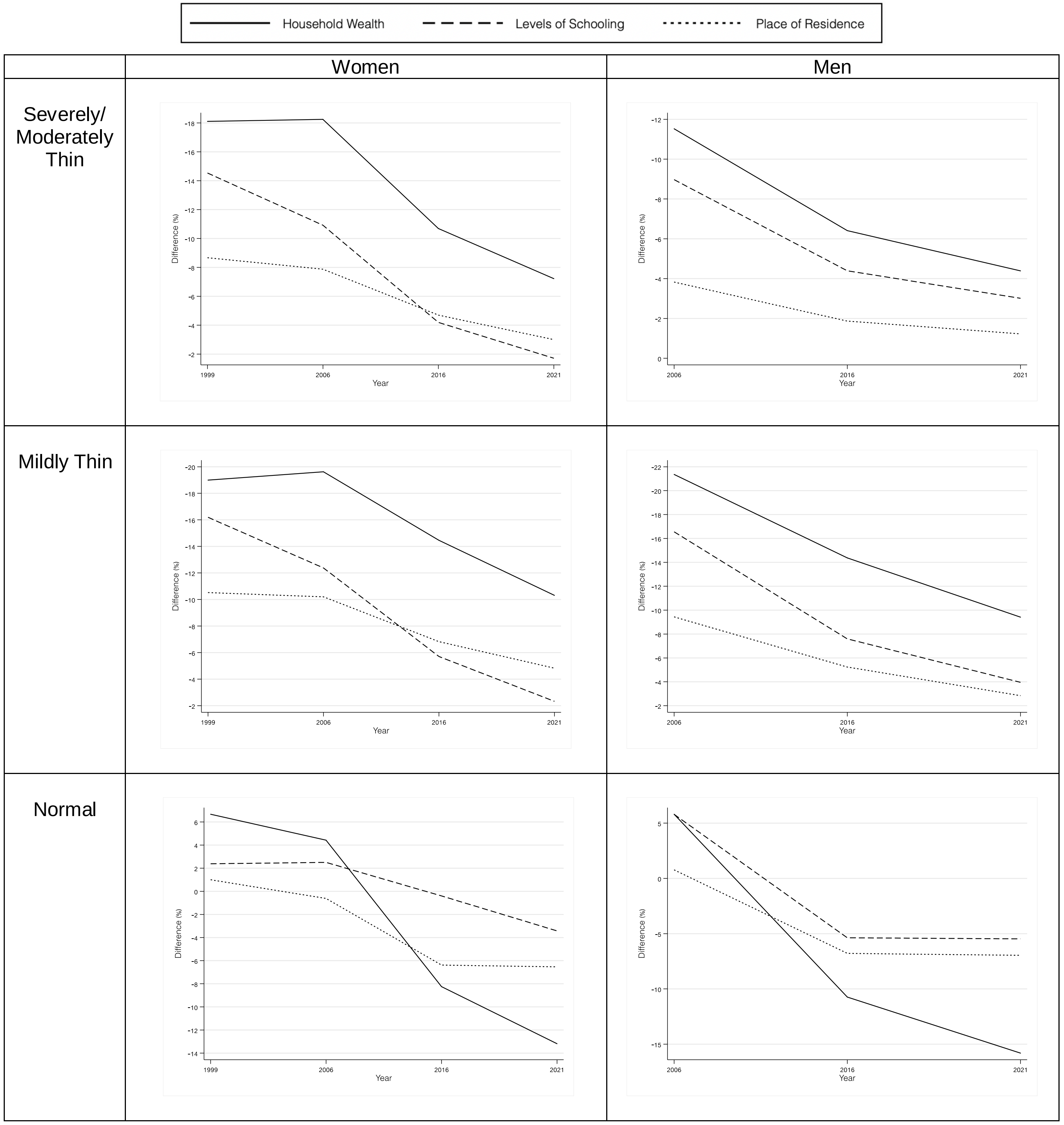

<table>
<tr>
<td>Overweight</td>
<td>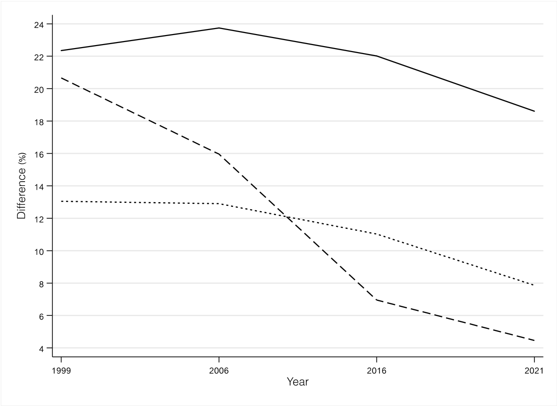
</td>
<td>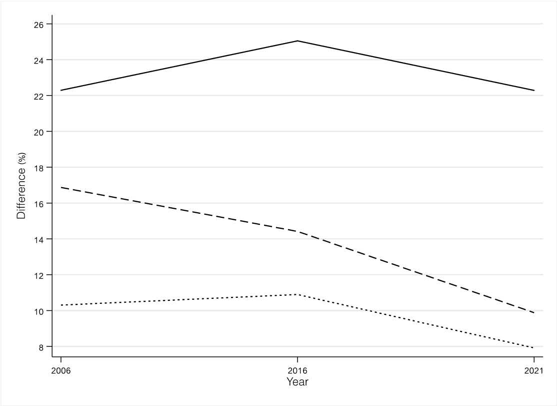
</td>
</tr>
<tr>
<td>Obese</td>
<td>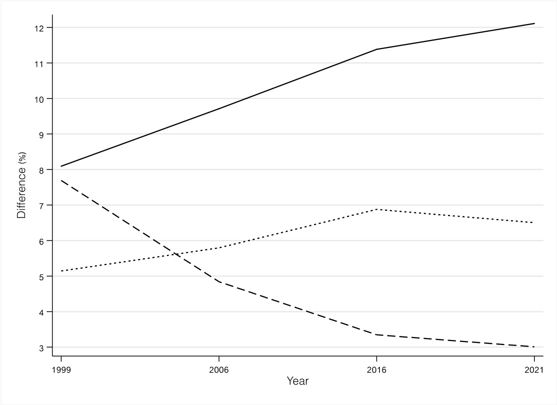
</td>
<td>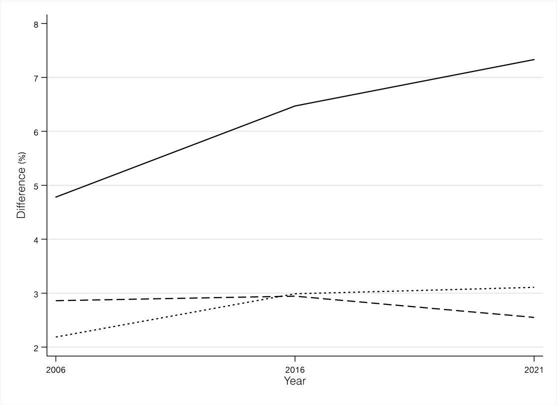
</td>
</tr>
</table>

Note: Difference in Wealth is calculated as ("Highest (Top 20%)" – "Lowest (bottom 20%)"). Difference in levels of schooling is calculated as (">12th grade" – "no schooling"). Difference in residence is calculated as ("Urban"- "Rural"). Y axis for prevalence of severely/moderately thin and mildy thin is reversed (upper is smaller negative value).

Figure S3: Difference in Difference of Prevalence of BMI Outcome by SES Categories and Year

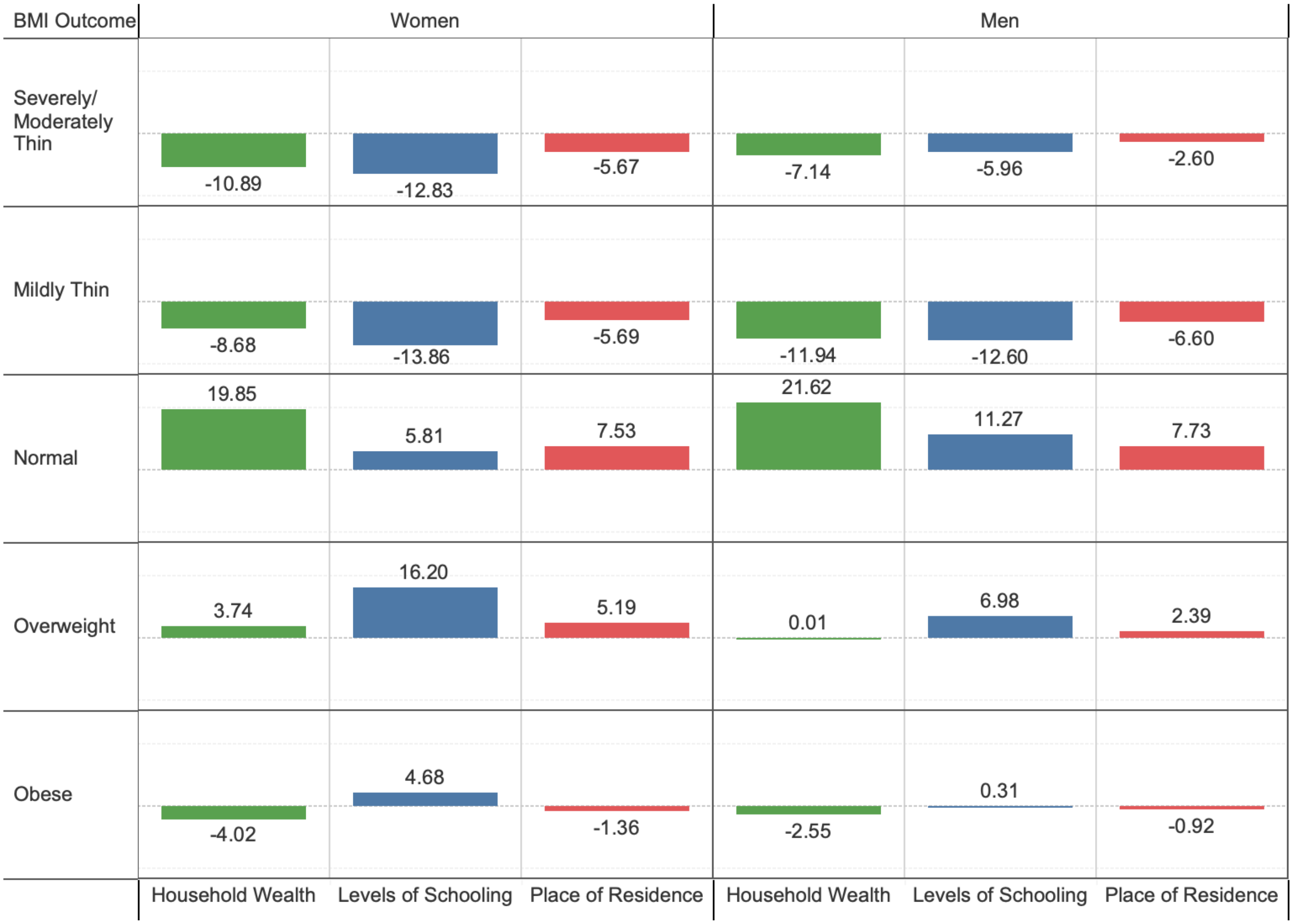

Note: The data shows the change in the difference of the prevalence of BMI Outcomes in the best off and worst off (highest wealth quintile – lowest wealth quintile, >12th grade – no schooling, urban rural). Changes over time was estimated by deducting value of most distant year from most recent year (2021). For men, the data was calculated by the difference in 2021 – difference in 2006. For women, it is the difference in 2021 – difference in 1999. A negative value indicates that the gap between the best off and worst off has decreased over time.

Appendix S1: Bibliography of the studies found from a systematic search and reviewed as a part of our st udy

1. Adlakha D, Hipp JA, Brownson RC. Neighborhood-based differences in walkability, physical activity, and weight status in India. Journal of Transport & Health. Dec 2016;3(4):485-499. doi:10.1016/j.jth.2016.10.008
2. Ali MK, Bhaskarapillai B, Shivashankar R, et al. Socioeconomic status and cardiovascular risk in urban South Asia: The CARRS Study. Eur J Prev Cardiol. Mar 2016;23(4):408-19. doi:10.1177/2047487315580891
3. Ambade M, Kim R, Subramanian SV. Socio-economic distribution of modifiable risk factors for cardiovascular diseases: An analysis of the national longitudinal ageing study in India. Prev Med. Oct 2023;175:107696. doi:10.1016/j.ypmed.2023.107696
4. Basu S, Sidh SN. Work status and health of women: a comparative study of northern and southern states of rural India. World Health Popul. 2008;10(2):40-52. doi:10.12927/whp.2008.19930
5. Bhan N, Millett C, Subramanian SV, et al. Socioeconomic patterning of chronic conditions and behavioral risk factors in rural South Asia: a multi-site cross-sectional study. International Journal of Public Health. Dec 2017;62(9):1019-1028. doi:10.1007/s00038-017-1019-9
6. Bharati P, Bharati S, Pal M, Chakraborty S, Gupta R. Chronic energy deficiency among Indian women by residential status. Ecology of Food and Nutrition. 2008;47(2):170-187. doi:10.1080/03670240701703055
7. Bharati S, Pal M, Battacharya BN, Bharati P. Prevalence and causes of chronic energy deficiency and obesity in Indian women. Human Biology. Aug 2007;79(4):395-412. doi:10.1353/hub.2007.0048
8. Bharati S, Pal M, Sen S, Bharati P. Malnutrition and anaemia among adult women in India. Journal of Biosocial Science. Sep 2019;51(5):658-668. Pii s002193201800041x. doi:10.1017/s002193201800041x
9. Chhabra P, Chhabra SK. Distribution and determinants of body mass index of non-smoking adults in Delhi, India. J Health Popul Nutr. Sep 2007;25(3):294-301.
10. Dutta M, Selvamani Y, Singh P, Prashad L. The double burden of malnutrition among adults in India: evidence from the National Family Health Survey-4 (2015-16). Epidemiology and Health. Dec 2019;41e2019050. doi:10.4178/epih.e2019050
11. Ganie MA, Chowdhury S, Suri V, et al. Prevalence, Regional Variations, and Predictors of Overweight, Obesity, and Hypertension Among Healthy Reproductive-Age Indian Women: Nationwide Cross-Sectional Polycystic Ovary Syndrome Task Force Study. JMIR Public Health Surveill. Sep 6 2023;9:e43199. doi:10.2196/43199
12. Geldsetzer P, Manne-Goehler J, Theilmann M, et al. Geographic and sociodemographic variation of cardiovascular disease risk in India: A cross-sectional study of 797,540 adults. Plos Medicine. Jun 2018;15(6)e1002581. doi:10.1371/journal.pmed.1002581
13. Gouda J, Prusty RK. Overweight and Obesity among Women by Economic Stratum in Urban India. Journal of Health Population and Nutrition. Mar 2014;32(1):79-88.
14. Gupta R, Deedwania PC, Sharma K, et al. Association of Educational, Occupational and Socioeconomic Status with Cardiovascular Risk Factors in Asian Indians: A Cross-Sectional Study. Plos One. Aug 2012;7(8)e44098. doi:10.1371/journal.pone.0044098
15. Gupta R, Guptha S, Gupta VP, Agrawal A, Gaur K, Deedwania PC. Twenty-year trends in cardiovascular risk factors in India and influence of educational status. Eur J Prev Cardiol. Dec 2012;19(6):1258-71. doi:10.1177/1741826711424567
16. Hu P, Wang S, Lee J. Socioeconomic gradients of cardiovascular risk factors in China and India: results from the China health and retirement longitudinal study and longitudinal aging study in India. Int J Public Health. Sep 2017;62(7):763-773. doi:10.1007/s00038-017-0968-3
17. Kumar P, Mangla S, Kundu S. Inequalities in overweight and obesity among reproductive age group women in India: evidence from National Family Health Survey (2015-16). Bmc Womens Health. Jun 2022;22(1)205. doi:10.1186/s12905-022-01786-y
18. Little M, Humphries S, Patel K, Dewey C. Factors associated with BMI, underweight, overweight, and obesity among adults in a population of rural south India: a cross-sectional study. BMC Obes. 2016;3:12. doi:10.1186/s40608-016-0091-7

19. Luhar S, Mallinson PAC, Clarke L, Kinra S. Trends in the socioeconomic patterning of overweight/obesity in India: a repeated cross-sectional study using nationally representative data. Bmj Open. Oct 2018;8(10)e023935. doi:10.1136/bmjopen-2018-023935
20. Meshram, II, Balakrishna N, Sreeramakrishna K, et al. Trends in nutritional status and nutrient intakes and correlates of overweight/obesity among rural adult women (18-60 years) in India: National Nutrition Monitoring Bureau (NNMB) national surveys. Public Health Nutrition. Apr 2016;19(5):767-776. doi:10.1017/s1368980015002268
21. Ogunsina K, Dibaba DT, Akinyemiju T. Association between life-course socio-economic status and prevalence of cardio-metabolic risk ractors in five middle-income countries. J Glob Health. Dec 2018;8(2):020405. doi:10.7189/jogh.08.020405
22. Pengpid S, Peltzer K. Prevalence and correlates of underweight and overweight/obesity among women in India: results from the National Family Health Survey 2015-2016. Diabetes Metabolic Syndrome and Obesity-Target & Therapy. 2019;12:647-653. doi:10.2147/dmso.S206855
23. Pengpid S, Peltzer K. Underweight and overweight/obesity among middle aged and older adults in India: Prevalence and correlates from a national survey in 2017-2018. International Journal of Noncommunicable Diseases. Oct-Dec 2021;6(4):172-179. doi:10.4103/jncd.jncd_9_21
24. Rai RK, Jaacks LM, Bromage S, Barik A, Fawzi WW, Chowdhury A. Prospective cohort study of overweight and obesity among rural Indian adults: sociodemographic predictors of prevalence, incidence and remission. Bmj Open. Aug 2018;8(8)e021363. doi:10.1136/bmjopen-2017-021363
25. Reddy BN. Body mass index and its association with socioeconomic and behavioral variables among socioeconomically heterogeneous populations of Andhra Pradesh, India. Human Biology. Oct 1998;70(5):901-917.
26. Rengma MS, Sen J, Mondal N. Socio-Economic, Demographic and Lifestyle Determinants of Overweight and Obesity among Adults of Northeast India. Ethiop J Health Sci. Jul 2015;25(3):199-208. doi:10.4314/ejhs.v25i3.2
27. Saha A, Muhammad T, Mandal B, Adhikary M, Barman P. Socio-demographic and behavioral correlates of excess weight and its health consequences among older adults in India: Evidence from a cross-sectional study, 2017-18. PLoS One. 2023;18(10):e0291920. doi:10.1371/journal.pone.0291920
28. Samal S, Panigrahi P, Dutta A. Social epidemiology of excess weight and central adiposity in older Indians: analysis of Study on global AGEing and adult health (SAGE). Bmj Open. 2015;5(11)e008608. doi:10.1136/bmjopen-2015-008608
29. Sethi V, de Wagt A, Bhanot A, et al. Levels and determinants of malnutrition among India's urban poor women: An analysis of Demographic Health Surveys 2006 and 2016. Maternal and Child Nutrition. Jul 2020;16(3)e12978. doi:10.1111/mcn.12978
30. Siddiqui MZ, Donato R. Overweight and obesity in India: policy issues from an exploratory multi-level analysis. Health Policy and Planning. Jun 2016;31(5):582-591. doi:10.1093/heapol/czv105
31. Siddiqui MZ, Donato R. Undernutrition among adults in India: the significance of individual-level and contextual factors impacting on the likelihood of underweight across sub-populations. Public Health Nutrition. Jan 2017;20(1):130-141. doi:10.1017/s1368980016001968
32. Singh RB, Beegom R, Mehta AS, et al. Social class, coronary risk factors and undernutrition, a double burden of diseases, in women during transition, in five Indian cities. International Journal of Cardiology. May 1999;69(2):139-147. doi:10.1016/s0167-5273(99)00010-8
33. Subramanian SV, Kawachi I, Smith GD. Income inequality and the double burden of under- and overnutrition in India. Journal of Epidemiology and Community Health. Sep 2007;61(9):802-809. doi:10.1136/jech.2006.053801
34. Subramanian SV, Perkins JM, Khan KT. Do burdens of underweight and overweight coexist among lower socioeconomic groups in India? American Journal of Clinical Nutrition. Aug 2009;90(2):369-376. doi:10.3945/ajcn.2009.27487
35. Verma M, Das M, Sharma P, Kapoor N, Kalra S. Epidemiology of overweight and obesity in Indian adults - A secondary data analysis of the National Family Health Surveys. Diabetes Metab Syndr. Jul-Aug 2021;15(4):102166. doi:10.1016/j.dsx.2021.06.003

36. Young MF, Nguyen [60] P, Tran LM, Avula R, Menon P. A Double Edged Sword? Improvements in Economic Conditions over a Decade in India Led to Declines in Undernutrition as Well as Increases in Overweight among Adolescents and Women. Journal of Nutrition. Feb 2020;150(2):364-372. doi:10.1093/jn/nxz251
37. Zaman MJ, Patel A, Jan S, et al. Socio-economic distribution of cardiovascular risk factors and knowledge in rural India. International Journal of Epidemiology. Oct 2012;41(5):1302-1314. doi:10.1093/ije/dyr226

Appendix S2: National Family Health Survey (NFHS): Summary of Survey Design

NFHS-2 (1998-1999) and NFHS-3 (2005-2006)

The NFHS-2 sample covers more than 99 percent of India's population living in all 26 states. It does not cover the union territories. NFHS-2 is a household survey with an overall target sample size of approximately 90,000 ever-married women in the age group 15–49. The target sample size was set considering the size of the state, the resources available for the survey, and the aggregate level (urban/rural, region, metropolitan cities) at which separate estimates were needed. The initial target sample size was 4,000 completed interviews with eligible women in states with a 1991 population of more than 25 million, 3,000 completed interviews with eligible women in states with a 1991 population between 2 and 25 million, and 1,500 completed interviews with eligible women in states with a population of less than 2 million. A uniform sample design was adopted in all the states. In each state, the rural sample was selected in two stages: the selection of Primary Sampling Units (PSUs), which are villages, with probability proportional to population size (PPS) at the first stage, followed by the random selection of 9 households within each PSU in the second stage. In urban areas, a three-stage procedure was followed. In the first stage, wards were selected with PPS sampling. In the next stage, one census enumeration block (CEB) was randomly selected from each sample ward (except in Jammu and Kashmir, where two CEBs were randomly selected from each sample ward). In the final stage, households were randomly selected within each sample CEB. In NFHS 3, the sampling frame was census 2001 data.

NFHS-4 (2015-2016) and NFHS-5 (2019-2021)

NFHS-4 and NFHS-5 are designed to provide estimates at district levels. The sample is a stratified two-stage sample. The 2011 census served as the sampling frame for the selection of PSUs. PSUs were villages in rural areas and Census Enumeration Blocks (CEBs) in urban areas. PSUs with fewer than 40 households were linked to the nearest PSU. Within each rural stratum, villages were selected from the sampling frame with probability proportional to size (PPS). In each stratum, six approximately equal substrata were created by crossing three substrata, each created based on the estimated number of households in each village, with two substrata, each created based on the percentage of the population belonging to scheduled castes and scheduled tribes (SCs/STs). Within each explicit sampling stratum, PSUs were sorted according to the literacy rate of women age 6+ years. The final sample PSUs were selected with PPS sampling. In urban areas, CEB information was obtained from the Office of the Registrar General and Census Commissioner, New Delhi. CEBs were sorted according to the percentage of the SC/ST population in each CEB, and sample CEBs were selected with PPS sampling. In every selected rural and urban PSU, a complete household mapping and listing operation was conducted prior to the main survey. Selected PSUs with an estimated number of at least 300 households were segmented into segments of approximately 100-150 households. Two of the segments were randomly selected for the survey using systematic sampling with probability proportional to segment size. Therefore, an NFHS-4 cluster is either a PSU or a segment of a PSU. In the second stage, in every selected rural and urban cluster, 22 households were randomly selected with systematic sampling.
